# From Cortex to Cardiac Response: tDCS of the Prefrontal Cortex Improves Autonomic Markers of Emotion Regulation

**DOI:** 10.3390/brainsci15090898

**Published:** 2025-08-22

**Authors:** Catarina Gomes Coelho, Jorge Leite, Raquel Pinto, Paulo P. P. Machado, Sandra Carvalho

**Affiliations:** 1 Psychological Neuroscience Laboratory, Centro de Investigação em Psicologia (CIPsi), Department of Basic Psychology, School of Psychology, University of Minho, 4710-057 Braga, Portugal; id9324@alunos.uminho.pt; 2 Psychotherapy and Psychopathology Research Laboratory, Centro de Investigação em Psicologia (CIPsi), School of Psychology, University of Minho, 4710-057 Braga, Portugal; pmachado@psi.uminho.pt; 3 CINTESIS@RISE, CINTESIS.UPT, Portucalense University, 4200-072 Porto, Portugal; jorgel@upt.pt; 4 Department of Education and Psychology, University of Aveiro, 3810-198 Aveiro, Portugal; raquelspinto@ua.pt

**Keywords:** emotion regulation, cognitive reappraisal, transcranial direct current stimulation, heart rate, skin conductance, respiratory rate

## Abstract

**Background:** Emotion regulation (ER) plays a vital role in mental health, spanning mood, anxiety, and personality disorders. Cognitive reappraisal (CR) is one of the most common ER strategies and depends on prefrontal brain areas, but its success varies, and its neural basis is not fully clear. Interest is growing in using transcranial direct current stimulation (tDCS) to support ER, yet most studies have focused only on the dorsolateral prefrontal cortex (dlPFC) and used simple tasks. **Objective:** This study explored whether tDCS applied to either the dlPFC or the ventromedial prefrontal cortex (vmPFC) could shape autonomic responses during CR while people watched emotionally engaging film clips. **Methods:** Participants were randomly assigned to receive either active or sham tDCS over the dlPFC or vmPFC. While stimulated, they used CR strategies (positive reappraisal, fictional reappraisal, or distancing) to manage their reactions to negative film scenes. Heart rate (HR), skin conductance (SC), and respiratory rate (RR) were tracked throughout as physiological indicators. **Results:** Active dlPFC tDCS combined with CR led to significantly greater reductions in HR toward the end of emotional exposure, compared to sham or non-CR conditions. dlPFC stimulation also lowered HR even without explicit CR, pointing to possible effects on automatic regulation. vmPFC effects were inconsistent, and no reliable effects were observed for SC or RR. **Conclusions:** These results suggest that tDCS effects on autonomic ER depend on the brain region and timing. dlPFC stimulation may strengthen both intentional and automatic emotion regulation, especially when combined with reappraisal, highlighting the value of realistic emotional tasks in neuromodulation studies.

## 1. Introduction

Emotion regulation (ER) is a fundamental component of mental health and psychological resilience, encompassing the processes individuals use to modify their emotional experiences, expressions, and related physiological responses based on contextual demands [1]. ER difficulties are transdiagnostic—spanning mood, anxiety, and personality disorders—and are consistently linked to functional impairment and diminished well-being [2,3]. Cognitive reappraisal (CR) is among the most effective and versatile strategies, involving reframing the interpretation of an affect-eliciting situation to attenuate its emotional impact [4].

CR has various subtypes, such as positive reappraisal (emphasizing the advantages of negative circumstances), fictional reappraisal (reinterpreting emotionally impactful fictional narratives), and distancing (assuming a detached or third-person viewpoint) [5,6]. These strategies assist individuals in deliberately diminishing unpleasant affect and fostering more adaptive emotional viewpoints. Nonetheless, individual disparities in the ability to execute CR efficiently may be affected by variations in cognitive resources and neurobiological functioning [7,8].

Functional neuroimaging research has delineated a network of cerebral areas implicated in cognitive ER, notably the dorsolateral prefrontal cortex (dlPFC) and the ventromedial prefrontal cortex (vmPFC) [9,10]. The dlPFC is mostly linked to working memory and the preservation of regulatory objectives [11]. On the other hand, the vmPFC is essential for synthesizing emotional information and assessing the affective relevance of inputs [12]. In cognitive control, these regions interact whereby the dlPFC is believed to exert a top-down influence on the vmPFC, which subsequently modulates activity in limbic structures, especially the amygdala [11,13]. However, their relative contributions across reappraisal strategies and the causal impact of directly modulating each region remain insufficiently characterized [14].

Psychophysiological indices, heart rate (HR), skin conductance (SC), and respiratory rate (RR), provide objective readouts of arousal and regulatory success. In general, higher autonomic activation indexes greater arousal [15], and CR has been linked to HR (and sometimes SC) reductions [16,17]. RR, though less studied, also covaries bidirectionally with emotion [18]. Beyond psychophysiology, peripheral biomarkers can index mechanisms linking prefrontal control and autonomic regulation: salivary markers of arousal/neuromodulatory tone (e.g., orexin-A) are sensitive to Non-invasive brain stimulation (NIBS) [19], circulating miRNAs may track CNS plasticity [20], and meta-analytic evidence shows NIBS elevates neurotrophic factors such as BDNF [21]. Taken together, these peripheral markers—alongside autonomic indices—motivate a causal approach to probe how prefrontal control shapes autonomic regulation.

NIBS techniques, especially transcranial direct current stimulation (tDCS), have arisen as effective methods for influencing emotional and cognitive functions by modifying cortical excitability [22]. Considering the pivotal function of the prefrontal cortex in ER, tDCS presents an intriguing approach for augmenting regulatory abilities via focused neuronal stimulation [23]. tDCS delivers a low-intensity direct current to targeted cortical sites, shifting membrane potentials in a polarity-dependent manner (anodal ↑ excitability, cathodal ↓ excitability) and modulating local activity and functional connectivity; the precise effects vary with montage and cortical target [21].

Growing evidence indicates that anodal dlPFC tDCS enhances executive functioning, strengthens top-down control, and reduces emotional reactivity [24,25]. These findings indicate that altering prefrontal activity may enhance the application of emotion management methods, including CR. However, most current research has focused primarily on the dlPFC, while only a few studies have investigated the effects of activating the vmPFC, which is essential for emotional appraisal and limbic modulation [26].

Direct comparisons of dlPFC versus vmPFC stimulation on multimodal outcomes remain rare, and few studies test their interaction with specific regulation strategies in ecologically valid paradigms (e.g., emotionally evocative film clips). Even fewer studies have investigated how the stimulation of these regions interacts with various cognitive regulation strategies or evaluated these interactions within ecologically valid paradigms, such as the utilization of emotionally evocative film clips, which more accurately reflect real-world emotional experiences than static images or hypothetical scenarios [27].

Therefore, there is a critical need to clarify how targeted neuromodulation interacts with distinct CR strategies to regulate emotional responses, particularly when assessed through psychophysiological measures in ecologically valid contexts, such as emotionally evocative film clips, which offer greater realism than static images.

Accordingly, we contrasted the dlPFC (executive control/goal maintenance) with the vmPFC (valuation/context integration and safety learning) to test whether neuromodulation preferentially augments top-down control during reappraisal. Our primary objective was to determine whether anodal tDCS to the dlPFC or vmPFC, combined with CR strategies, reduces autonomic arousal—indexed by HR, SC, and RR—while participants view emotionally negative film clips, relative to sham. The primary objective was to assess whether active tDCS (to the dlPFC or vmPFC), in combination with CR strategies (positive reappraisal, fictional reappraisal, or distancing), would lead to greater reductions in autonomic arousal compared to sham stimulation.

The study also pursued three secondary objectives. First, it aimed to evaluate target-specific effects by comparing the differential impact of dlPFC versus vmPFC stimulation on psychophysiological responses during CR. Second, it sought to examine subjective emotional responses by measuring changes in self-reported positive and negative affect using the Positive and Negative Affect Schedule (PANAS), and assessed perceived side effects with the Visual Analogue Scale (VAS) from pre- to post-intervention. Third, the study aimed to assess the effectiveness of blinding by evaluating participants’ beliefs regarding whether they received active or sham stimulation, along with their confidence in these judgments.

By pairing neuromodulation with multiple CR strategies and continuous psychophysiology in an ecologically valid paradigm, we aim to clarify mechanisms of ER and inform the potential clinical utility of tDCS.

## 2. Materials and Methods

### 2.1. Study Design

This study followed a randomized, single-blind, sham-controlled, within-subjects crossover design, incorporating a between-subjects variable for stimulation site (dlPFC versus vmPFC). Participants were randomly allocated to receive tDCS targeting either the dlPFC or the vmPFC. Each subject underwent two experimental sessions: one involving active tDCS and the other using sham stimulation. The sequence was counterbalanced and separated by a minimum interval of 48 h to prevent carryover effects [28].

**Figure 1:**
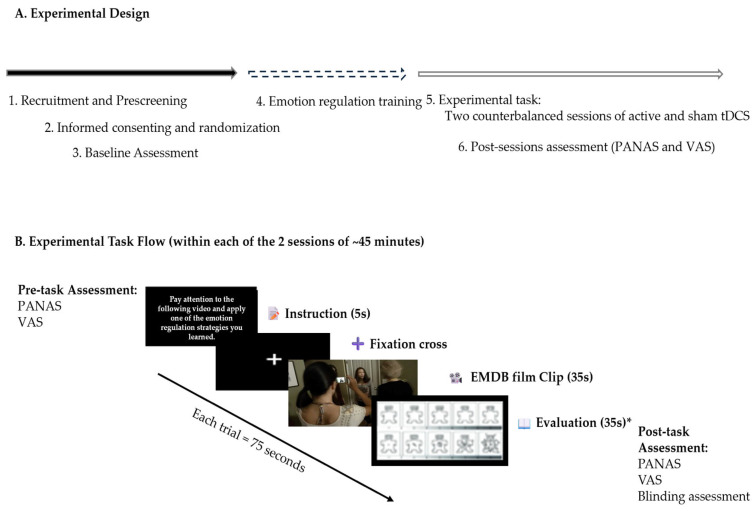
Overview of the experimental design: (**A**) Sequential stages of the study, including recruitment, baseline assessment, emotion regulation training, and two counterbalanced stimulation sessions (active and sham tDCS) targeting the dlPFC or vmPFC; (**B**) Structure of the experimental task during each session—The order of conditions, film clips, electrode placement, and stimulation type (active or sham) was randomized. * Note. Due to technical issues, the system failed to register responses from the Self-Assessment Manikin (valence and arousal), resulting in the loss of these data.

During each session, participants undertook a negative emotion induction task utilizing emotionally evocative film excerpts from the Emotional Movie Database (EMDB), while applying previously trained CR strategies. cognitive reappraisal techniques. Psychophysiological metrics (heart rate, skin conductance, and respiratory rate) were constantly monitored, while affective states were evaluated before and after stimulation utilizing the PANAS. This design enabled both within-subject comparisons (active versus sham stimulation) and between-group comparisons (dlPFC versus vmPFC stimulation), while accounting for individual variability and permitting a thorough investigation of psychophysiological, affective, and side effect results (Figure 1).

Randomization and blinding. Assignment to dlPFC vs. vmPFC used computer-generated permuted block randomization (variable block sizes 4 and 6; 1:1). No stratification factors were applied. The allocation list was prepared by an investigator independent of enrollment and data collection; device codes implemented active vs. sham and preserved allocation concealment and blinding of participants and experimenters. Within participants, session order (active/sham) was counterbalanced (AB/BA).

### 2.2. Ethical Approval

The study was reviewed and approved by the Ethics Committee for Research in Social and Human Sciences at the University of Minho (Approval No. CEICSH 060/2022). Data collection was conducted at the BrainLoop Neuroscience Laboratory of Universidade Portucalense—Infante D. Henrique, in Porto, Portugal.

### 2.3. Participants

Participants were randomly assigned to one of two experimental groups: dlPFC stimulation or vmPFC stimulation. Participants were eligible for inclusion if they were 18 years of age or older, native Portuguese speakers, and permanent residents of Portugal. These criteria ensured that participants had linguistic and cultural homogeneity, as well as the ability to comprehend and follow the study procedures. Exclusion criteria were defined to minimize potential risks associated with transcranial direct current stimulation (tDCS) and to control confounding variables. Individuals were excluded if they presented any known contraindications for tDCS application, such as the presence of metallic implants in the head (excluding the oral cavity) or implanted medical devices (e.g., pacemakers or neurostimulators). Additional exclusion criteria included a self-reported history of psychiatric or neurological disorders (e.g., epilepsy), illicit psychotropic substances use, and left-handedness, as determined by a score below 70 on the Edinburgh Handedness Inventory. The restriction to right-handed participants was implemented to reduce variability in cortical organization and lateralization effects that could influence stimulation outcomes [29].

Although 94 individuals were initially recruited, 9 participants were excluded: 7 for missing one session, 1 for completing the same version of the emotional induction task in both sessions, and 1 due to technical malfunction of the stimulator.

### 2.4. Assessments

#### 2.4.1. Eligibility Measures and Questionnaires

Sociodemographic and Clinical Questionnaire: This self-report questionnaire was developed to gather extensive background information, including participants’ age, sex, gender, nationality, educational level, employment position, and marital status. It also evaluated health-related behaviors like alcohol and tobacco use, other substance intake, and medical history—specifically concentrating on current or previous clinical problems and continuing pharmaceutical treatments. This data was utilized to profile the sample and identify potential confounding factors pertinent to neuromodulation safety and emotional processing.

tDCS Eligibility Assessment: a 16-item dichotomous (yes/no) tDCS eligibility questionnaire was used to screen for contraindications, including prior seizures, unexplained loss of consciousness, neurological conditions, serious head injury, metal implants (excluding dental fillings), implanted medical devices (e.g., pacemakers), pregnancy status, and current medication use. Participants also reported any prior exposure to or adverse reactions from tDCS. Items assessed both personal and family history (e.g., epilepsy), and participants could request additional information about the procedure and its risks. Those presenting any exclusion criteria were not enrolled.

Edinburgh Handedness Inventory (EHI; Espírito-Santo et al., 2017 [30]; originally developed by Oldfield, 1971 [31]), a 10-item self-report inventory that assesses hand preference in routine activities. Scores vary from −100 (indicating a strong left preference) to +100 (indicating a strong right preference). The Portuguese version exhibits strong internal consistency (Cronbach’s *α* = 0.88).

#### 2.4.2. Baseline Assessment Instruments 

To comprehensively characterize participants’ cognitive-emotional profiles, resilience, and personality traits, three standardized self-report instruments were administered during the initial assessment phase. All instruments used validated Portuguese versions with established psychometric properties.

Cognitive Emotion Regulation Questionnaire (CERQ; Martins et al., 2016 [32]; original version by Garnefski et al., 2001 [33]) is a 36-item self-report measure designed to assess individual differences in the use of cognitive ER strategies following negative or stressful life events. Items are rated on a 5-point Likert scale ranging from 1 (almost never) to 5 (almost always). The questionnaire is composed of nine subscales: Self-blame, Rumination, Catastrophizing, Blaming Others, Acceptance, Positive Reappraisal, Refocus on Planning, Positive Refocusing, and Putting into Perspective. The Portuguese version has demonstrated adequate internal consistency, with Cronbach’s alpha values ranging from 0.75 to 0.91 across subscales.

Emotion Regulation Profile-Revised (ERP-R) is a validated self-report instrument that assesses individual differences in the use and effectiveness of ER strategies across a range of emotionally evocative scenarios. It presents participants with brief hypothetical vignettes and asks them to rate how likely they are to respond using various regulatory strategies. The ERP-R captures both adaptive strategies (e.g., Cognitive Reappraisal, Acceptance, Problem-solving) and maladaptive strategies (e.g., Suppression, Rumination, Avoidance). Developed by Nelis et al. (2011) [34], the ERP-R has demonstrated robust psychometric properties, including good internal consistency, convergent validity with measures of emotional intelligence and well-being, and sensitivity to interindividual differences.

Resilience Scale for Adults (RSA; Pereira et al., 2017 [35]; original version by Hjemdal et al., 2011 [36]) was used to evaluate individual levels of psychological resilience. This instrument consists of 33 items distributed across six protective factors: Perception of Self, Planned Future, Social Competence, Family Cohesion, Social Resources, and Structured Style. Participants respond using a semantic differential scale, indicating where they fall between two opposing descriptors (e.g., “I feel unimportant—I feel valuable”). Higher scores reflect higher levels of resilience. The Portuguese version of the RSA has shown excellent psychometric properties, including strong internal consistency (Cronbach’s *α* = 0.90).

Brief Symptom Inventory (BSI; Canavarro, 1999 [37]; original version by Derogatis, 1993 [38]) is a standardized self-report tool consisting of 53 items, designed to evaluate psychiatric symptom patterns in both clinical and non-clinical groups. It assesses nine principal symptom dimensions—Somatization, Obsessive-compulsive Symptoms, Interpersonal Sensitivity, Depression, Anxiety, Hostility, Phobic Anxiety, Paranoid Ideation, and Psychoticism. Participants evaluate the degree of distress caused by each symptom over the preceding seven days with a 5-point Likert scale, ranging from 0 (“not at all”) to 4 (“extremely”). The BSI has undergone extensive validation and exhibits strong psychometric features, including adequate internal consistency, with Cronbach’s alpha values ranging from 0.71 to 0.85 across subscales.

#### 2.4.3. Experimental Task Instruments

A set of self-report instruments was administered during the experimental sessions to assess participants’ affective states, evaluate the tolerability of the transcranial direct current stimulation (tDCS), and verify the integrity of the double-blind procedure.

Positive and Negative Affect Schedule (PANAS; Galinha and Pais-Ribeiro, 2012 [39]; original version by Watson et al., 1988 [40]) was used to measure participants’ affective states before and after each experimental session. The instrument consists of 20 adjectives representing emotional states, divided equally into two subscales: Positive Affect (PA) and Negative Affect (NA). Each item is rated on a 5-point Likert scale ranging from 1 (“very slightly or not at all”) to 5 (“extremely”), based on how the participant feels at the present moment. Higher scores indicate greater levels of the respective affective dimensions. The Portuguese version of PANAS has demonstrated excellent internal consistency, with Cronbach’s alpha coefficients of 0.86 for PA and 0.89 for NA. 

Visual Analogue Scale (VAS) for tDCS Side Effects: to monitor potential adverse effects of tDCS, VAS was administered both before and immediately after each stimulation session. This self-report instrument combines visual and numeric elements, consisting of 10 items rated on a Likert-type scale ranging from 0 (“not at all”) to 10 (“extremely”). It assesses a range of symptoms commonly associated with non-invasive brain stimulation, including fatigue, anxiety, sadness, agitation, drowsiness, itching, headache, other types of pain, tingling sensations, and metallic taste. This measure helped ensure participant safety and tolerability of the stimulation protocol.

tDCS Blinding Questionnaire: to assess the effectiveness of the double-blind procedure, participants completed a blinding questionnaire at the end of each session. They were asked to indicate their best guess regarding the type of stimulation received (active, sham, or “don’t know”) and to rate their confidence in that judgment on a 5-point scale (“not at all” to “extremely confident”). This measure was used to evaluate the success of participant blinding and to rule out expectancy effects that could confound the interpretation of results.

### 2.5. Cognitive Reappraisal Training Session

Prior to participating in the experimental task, all subjects underwent a structured Cognitive Reappraisal (CR) training session aimed at acquainting them with the emotion management mechanisms employed in the study. The workshop presented three CR techniques—positive reappraisal, fictional reappraisal, and distancing—each based on modern emotional control theory. Participants were provided with theoretical explanations, sequential instructions, and guided practice exercises to ensure understanding and proper implementation of each method.

To enhance experiential learning and replicate the conditions of the primary task, participants observed a series of negative valance film clips (excluded from the experimental trials) and were directed to implement the CR strategies accordingly. Following the practice of each technique, participants assessed their comprehension and preparedness to advance. Only individuals who expressed confidence in independently implementing the techniques progressed to the experimental phase.

Additional information concerning the structure, instructional resources, and content of the training session is available in the Supplementary Materials (Figure S1—Emotion Regulation Training Guidelines).

### 2.6. Emotional Induction Task

The emotional induction task was designed to elicit negative emotional responses in a controlled laboratory setting using film clips. It was developed and implemented using e-Prime software (version 3) and consisted of a series of short film clips selected from the Emotional Movie Database (EMDB; [41]; Carvalho et al., submitted). These clips had been previously validated for emotional valence and arousal in the Portuguese population.

Each experimental session included 21 clips: 15 with negative emotional content (e.g., scenes evoking fear, sadness, or distress) and 6 with neutral content. The total duration of the task was approximately 26 min, including a training phase and the main task. Prior to the main task, participants completed a brief training phase using three negative clips. During this phase, they were instructed to apply one of three pre-trained cognitive reappraisal strategies—positive reappraisal, fictional reappraisal, or distancing to become familiar with the task structure and regulatory techniques.

For each trial, participants first received strategy instructions on screen for 5 s. This was followed by the presentation of a film clip lasting 30 s, after which they had an additional 35 s to evaluate their emotional experience using the Self-Assessment Manikin (SAM), a non-verbal pictorial scale measuring valence (pleasant–unpleasant) and arousal (calm—excited). The instruction preceding each clip varied randomly between passive observation and active application of a CR strategy. All clips were presented without sound to avoid auditory influence, and the sequence of clips was randomized across participants to reduce order effects.

**Figure 2:**
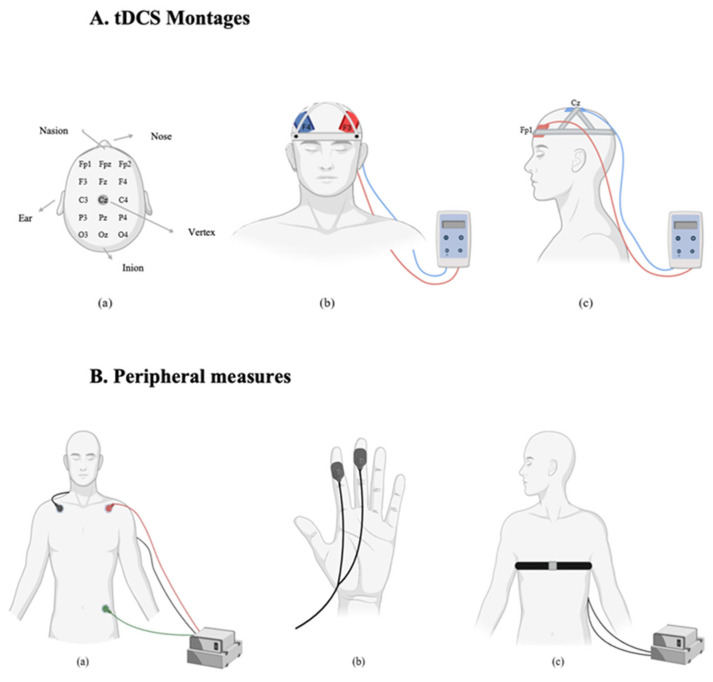
(**A**)**.** tDCS Montages: (**a**) Electrode placement based on the international 10–20 EEG system. (**b**) dlPFC stimulation montage: anodal electrode over F3 and cathodal electrode over F4 (bifrontal). (**c**) vmPFC stimulation montage: anodal electrode over Fp1 and cathodal electrode over Cz (frontal-midline). (**B**)**.** Peripheral Measures: (**a**) Electrocardiogram (ECG) setup for heart rate recording with three-lead chest configuration. (**b**) Electrodermal activity (EDA) measured via skin conductance sensors placed on the fingers. (**c**) Respiratory rate captured via thoracic belt transducer around the chest.

To minimize familiarity/habituation, we created two task versions (A/B); at the first session, participants were allocated by simple 1:1 randomization to start with Version A or B. These versions followed the same structure and distribution of emotional content but featured different film clips. Only the training phase contained the same stimuli across both versions. The structure of the task and the timing of each component are illustrated in Figure 2. Please refer to Table S1 in the Supplementary Material to assess the descriptions of the film clips used from the EMDB. Task versions.

### 2.7. Transcranial Direct Current Stimulation

Transcranial direct current stimulation (tDCS) was applied using the neuroConn DC-STIMULATOR PLUS (Germany). Electrode placements followed the 10–20 international EEG system [42]. Two sponge electrodes, each with an area of 35 cm^2^ (7 × 5 cm), were soaked in a 0.9% sodium chloride (NaCl) solution to improve conductivity and reduce skin resistance. Participants were randomly assigned to one of two stimulation montages. In the dlPFC group, a bifrontal montage was used. The anode was placed over the left dlPFC (F3) and the cathode over the right dlPFC (F4). In the vmPFC group, the anode was placed over the left vmPFC (Fp1) and the cathode over the vertex (Cz). In the active stimulation condition, a current of 2 mA was applied for 20 min. A 30 s ramp-up and 30 s ramp-down period was used to minimize discomfort. In the sham condition, the same electrode placements were used, but stimulation was applied for only 90 s (30 s ramp-up, 30 s at 2 mA, and 30 s ramp-down), after which the device was turned off automatically. This protocol ensured that participants experienced the initial sensations of stimulation, maintaining the integrity of the blinding procedure. Impedance was continuously monitored during each session to ensure it remained below 5 kΩ. Stimulation was only initiated when impedance levels were within this safe and acceptable range.

### 2.8. Peripheral Measures

Physiological data were recorded and processed using LabChart software (version 8). The processing steps were planned to minimize noise and artifacts and to isolate relevant signal components for subsequent analysis. These procedures are consistent with previous studies investigating the effects of tDCS on autonomic and emotional responses [25].

Heart rate (HR) data were cleaned to remove artifacts usually associated with movement or electrode noise. This was accomplished using the arithmetic option in LabChart, applying the function resample(Chi/4). This process reduces signal variability and improves the data smoothness. In the skin conductance data, the tonic component was isolated from the phasic component. To perform this, a new channel was created, and then, to clean the data, the following function was applied: Smoothsec(Differentiate(RClowpass(Ch5;0,205);3);1,3) in the arithmetic option. Respiratory rate (RR) data were recorded without additional processing, as the raw signal quality was deemed sufficient for the analysis.

Following signal cleaning, all physiological data were segmented according to the stimuli: neutral and negative (medium and high arousal) films. Data was extracted at fixed timepoints of 0–5, 5–10, 10–15, 15–20, 20–25 and 25–30 s for each film category. These timepoints were selected to capture temporal dynamics of physiological activity during emotional induction, as usually implemented in other studies [43].

### 2.9. Statistical Analyses

All analyses were performed in R Studio (version 4.5.0), with the significance threshold set at *p* < 0.05. To confirm that the two main stimulation groups (dlPFC and vmPFC) were comparable on sociodemographic and clinical variables, chi-square tests were used for categorical data and independent samples *t*-tests for continuous data. Within each stimulation group, participants were also categorized according to the presence or absence of emotion regulation (ER) difficulties, based on validated cut-off scores from the rumination subscale of the CERQ (≥14 for females; ≥12 for males).

Physiological responses (heart rate [HR], skin conductance [SC], and respiratory rate [RR]) were analyzed using a hierarchical approach. Each stimulation group (dlPFC or vmPFC) was subdivided into four experimental conditions: (1) active tDCS with cognitive reappraisal (CR); (2) active tDCS without CR; (3) sham tDCS with CR; and (4) sham tDCS without CR.

To test for regional and condition-specific effects, two-way mixed-design ANOVAs were conducted with stimulation region (dlPFC vs. vmPFC) as the between-subjects factor and condition as the within-subjects factor. For time-series analyses, the emotional film clips were segmented into six consecutive 5 s intervals (0–5 s, 5–10 s, 10–15 s, 15–20 s, 20–25 s, and 25–30 s) to capture the temporal dynamics of physiological responses. This binning approach follows prior work on the temporal unfolding of autonomic reactions to affective stimuli and helps isolate early versus late-phase effects.

To account for baseline autonomic variability, we indexed stimulus-evoked change using difference scores computed as simple subtraction (negative−neutral) for each 5 s bin. We chose subtraction over residualized change to preserve units and avoid additional model assumptions; all inferential analyses used these delta scores. These scores were analyzed with three-way mixed-design ANOVAs including stimulation site (between-subjects), condition, and time interval (within-subjects), and separate within-group repeated-measures ANOVAs further explored condition and time effects for dlPFC and vmPFC groups individually.

Effect sizes were calculated using partial eta squared (*η*^2^*_p_*) and Cohen’s *d*. Where applicable, post hoc pairwise comparisons were corrected for multiple testing using Tukey’s HSD; all significant findings are reported with an indication of whether they survived correction. Non-significant trends (*p* values between 0.05 and 0.10) are noted but interpreted cautiously as exploratory.

For within-subject factors with more than two levels (Condition: 4; Time: 6) and for any interactions involving these factors-sphericity was assessed with Mauchly’s test. When violated, Greenhouse–Geisser corrections were applied; we report FGG with corrected degrees of freedom and the *ε* estimate. When sphericity held (*p* ≥ 0.05), uncorrected degrees of freedom are reported.

For subjective measures (PANAS) and side effects (VAS), pre–post changes within each session (active vs. sham) were examined using paired-samples *t*-tests. Independent samples *t*-tests compared active and sham conditions at each timepoint. Missing data were minimal (<5%) and handled with pairwise deletion.

If no formal power analysis was conducted, this is acknowledged as a limitation, as the moderate sample size may limit sensitivity to detect small effects in autonomic measures.

## 3. Results

### 3.1. Sociodemographic and Clinical Data

Eighty-five volunteers participated (*M*_age = 27.7 years, *SD* = 10.8; 81.2% female), divided into dlPFC (*n* = 46) and vmPFC (*n* = 39) groups. The groups did not differ significantly in age, sex, nationality, marital status, education, or occupation, confirming comparable baseline characteristics (Table 1). Approximately 42% reported difficulties in emotion regulation (ER).

Participants with ER difficulties in both groups showed significantly higher scores on maladaptive strategies (e.g., self-blame, rumination, catastrophizing; all *p* < 0.01) than those without difficulties. Notably, they also reported greater use of some adaptive strategies (e.g., acceptance, positive refocusing; *p* < 0.05).

Regarding mental health, participants with ER difficulties had higher overall symptom scores (dlPFC: *p* < 0.05) and higher scores on specific subscales (interpersonal sensitivity, anxiety, hostility, paranoid ideation; all *p* < 0.05). The vmPFC group showed higher psychoticism (*p* < 0.05). Lower family cohesion was also observed among vmPFC participants with ER difficulties (*p* < 0.05).

### 3.2. Physiological Responses (Full Clip)

For the full-clip analyses, usable data were HR: *n* = 85; SC: *n* = 82; RR: *n* = 59 (after artifact rejection and exclusion of missing channels). Reported degrees of freedom reflect these Ns and, where sphericity was violated, Greenhouse–Geisser corrections.

#### 3.2.1. Total Mean of Heart Rate 

**Figure 3:**
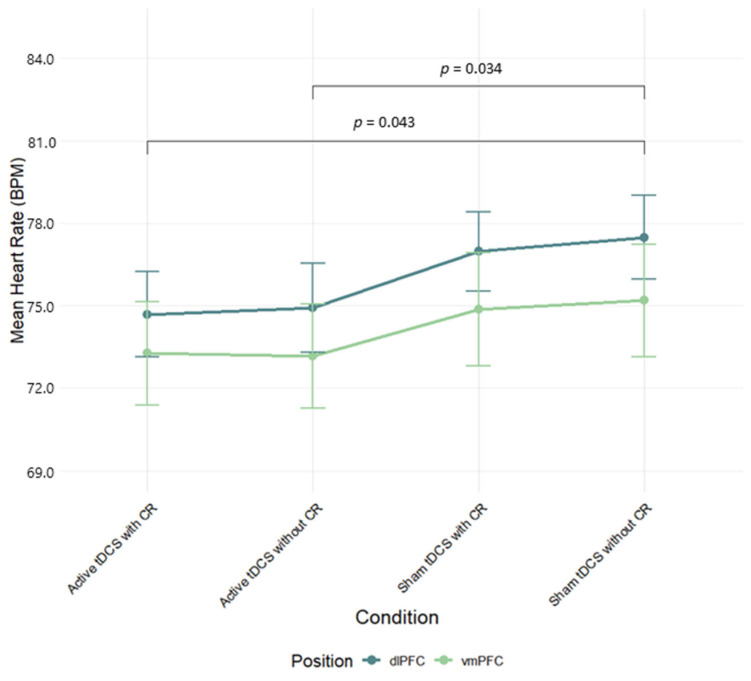
Mean heart rate (BPM) during negative film exposure for each condition, shown separately for dlPFC and vmPFC stimulation groups and conditions. Error bars show ±1 SEM.

A two-way ANOVA was performed to evaluate the effects of stimulation region (dlPFC, vmPFC) and condition (Active tDCS with CR, Active tDCS without CR, Sham tDCS with CR, Sham tDCS without CR) on heart rate (HR) mean scores. The means and standard deviations for HR response are presented in Table S2 in the Supplementary Material. The results indicated significant main effect of condition, *F*(3, 249) = 4.08, *p* = 0.008, *η*^2^*_p_* = 0.05; no significant main effect for stimulation region, *F*(1, 83) = 0.72, *p* = 0.40, *η*^2^*_p_* = 0.01; and no significant interaction between stimulation region and condition, *F*(3, 249) = 0.10, *p* = 0.96, *η*^2^*_p_* = 0.001 (Figure 3). Post hoc testing using Tukey’s HSD indicated that HR mean scores were significantly higher for sham tDCS without CR condition than for both active tDCS conditions, active with CR (*d* = 0.212, *p* = 0.034) and active without CR (*d* = 0.202, *p* = 0.043) (see Table S3 for full effect sizes).

#### 3.2.2. Total Mean of Skin Conductance 

**Figure 4:**
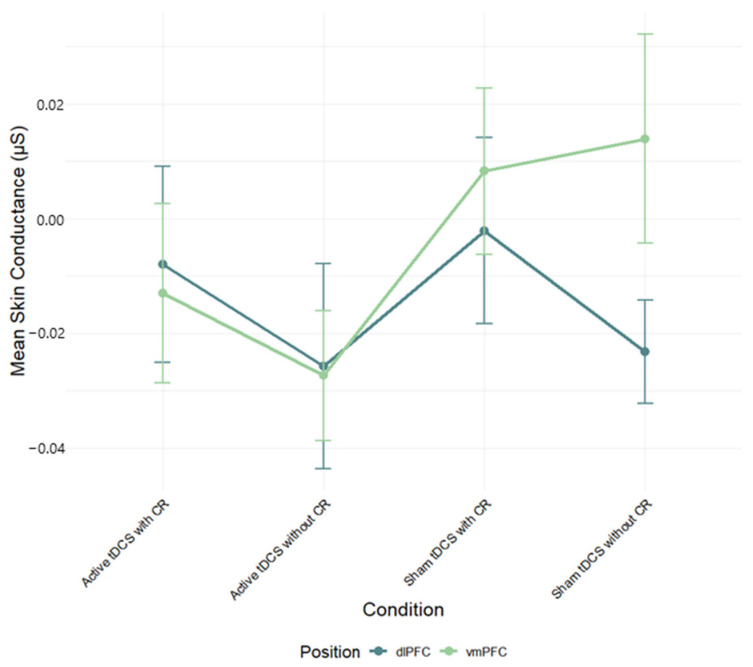
Mean skin conductance (μS) during negative film exposure for each condition, shown separately for dlPFC and vmPFC stimulation groups and conditions. Error bars show ±1 SEM.

A two-way ANOVA was performed to evaluate the effects of stimulation region and condition on skin conductance (SC) mean scores. The means and standard deviations for SC response are presented in Table S2 in the Supplementary Material. The results indicated no significant main effect for stimulation region, *F*(1, 82) = 0.68, *p* = 0.41, *η*^2^*_p_* = 0.01, condition, *F*(3, 240) = 1.34, *p* = 0.263, *η*^2^*_p_* = 0.02, and interaction between stimulation region and condition, *F*(3, 240) = 0.76, *p* = 0.519, *η*^2^*_p_* = 0.01 (Figure 4; see Table S4 for full effect sizes).

#### 3.2.3. Total Mean of Respiratory Rate

**Figure 5:**
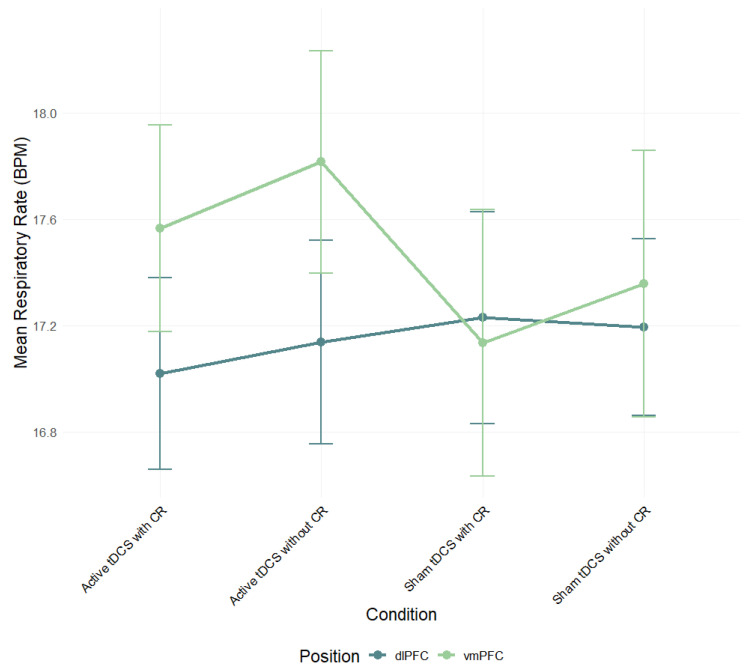
Mean respiratory rate (BPM) during negative film exposure for each condition, shown separately for dlPFC and vmPFC stimulation groups and conditions. Error bars show ±1 SEM.

A two-way ANOVA was performed to evaluate the effects of stimulation region and condition on respiratory rate (RR) mean scores. The means and standard deviations for RR response are presented in Table S2 in the Supplementary Material. The results indicated no significant main effect for stimulation region, *F*(1, 82) = 0.35, *p* = 0.556, *η*^2^*_p_* = 0.004, condition, *F*(3, 245) = 0.63, *p* = 0.594, *η*^2^*_p_* = 0.01, and interaction between stimulation region and condition, *F*(3, 245) = 2.06, *p* = 0.106, *η*^2^*_p_* = 0.02 (Figure 5; see Table S5 for full effect sizes).

### 3.3. Differences in Physiological Activation Across Time (5 s-Epochs)

#### 3.3.1. Heart Rate

**Figure 6:**
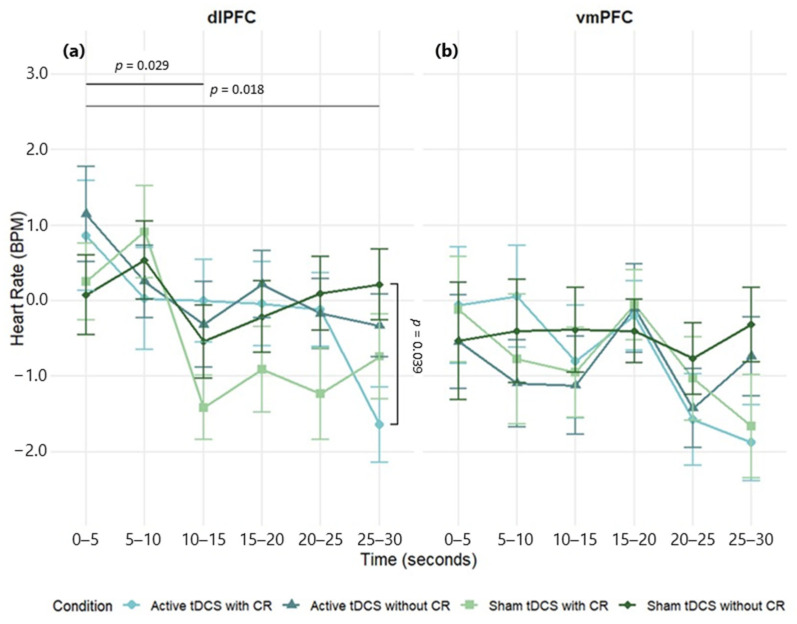
Mean heart rate change (BPM) over time during negative film exposure for each condition, shown separately for dlPFC (**a**) and vmPFC (**b**) stimulation groups. Heart rate values reflect difference scores (negative−neutral) across 5 s intervals. Error bars show ±1 SEM.

A three-way mixed-design ANOVA was conducted to examine the effects of stimulation region (dlPFC, vmPFC), condition (Active tDCS with CR, Active tDCS without CR, Sham tDCS with CR, Sham tDCS without CR), and time (0–5, 5–10, 10–15, 15–20, 20–25, 25–30 s) on heart rate (HR) difference scores. There was a significant main effect of time, *F*(5, 415) = 3.91, *p* = 0.002, *η*^2^*_p_* = 0.05. The main effects of stimulation region, *F*(1, 83) = 2.66, *p* = 0.107, *η*^2^*_p_* = 0.03, and condition, *F*(3, 249) = 0.71, *p* = 0.546, η^2^*_p_* = 0.01, were not significant. All interactions were non-significant: Region × Condition, *F*(3, 249) = 0.51, *p* = 0.674, *η*^2^*_p_* = 0.01; Region × Time, *F*(5, 415) = 0.91, *p* = 0.474, *η*^2^*_p_* = 0.01; Condition × Time, *F*(15, 1245) = 1.60, *p* = 0.066, *η*^2^*_p_* = 0.02; Region × Condition × Time, *F*(15, 1245) = 0.85, *p* = 0.62, *η*^2^*_p_* = 0.01. Although non-significant, dlPFC stimulation produced larger HR changes than vmPFC over time (Figure 6; Table S6).

In the dlPFC group, a significant main effect of time was observed, *F*(5, 225) = 2.87, *p* = 0.016, *η*^2^*_p_* = 0.06, indicating HR changes across the stimulus exposure. However, the main effect of condition was not significant, *F*(3, 135) = 1.06, *p* = 0.367, *η*^2^*_p_* = 0.02. A significant condition × time interaction was found, *F*(15, 675) = 1.84, *p* = 0.027, *η*^2^*_p_* = 0.04, suggesting that the experimental conditions differentially impacted HR responses over time. Post hoc comparisons revealed significant reductions in HR at 10–15 s (*d* = 0.30, *p* = 0.029) and 25–30 s (*d* = 0.32, *p* = 0.018) compared to the 0–5 s timepoint. At the 25–30 s point, the combination of active tDCS and CR decreased significantly the HR, when compared to sham tDCS without CR (*d* = 0.57, *p* = 0.039) (Figure 6a; see Table S7 for full effect sizes).

In the vmPFC group, the main effects of time, *F*(5, 190) = 2.02, *p* = 0.078, *η*^2^*_p_* = 0.05, condition, *F*(3, 114) = 0.26, *p* = 0.856, *η*^2^*_p_* = 0.01, and the condition × time interaction, *F*(15, 570) = 0.79, *p* = 0.692, *η*^2^*_p_* = 0.02, were not significant. However, descriptively, lower HR was observed for the active tDCS with CR and sham tDCS with CR conditions compared to others (Figure 6b; see Table S8 for full effect sizes).

#### 3.3.2. Skin Conductance

**Figure 7:**
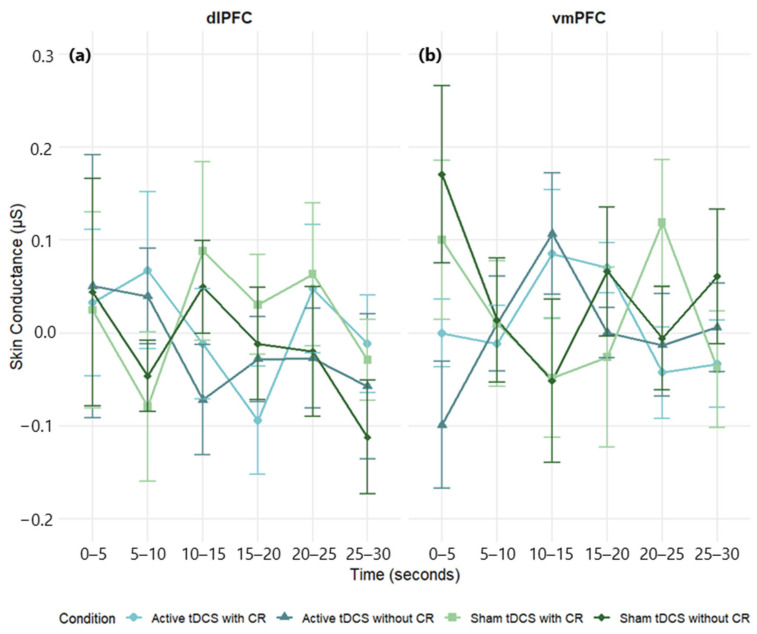
Mean skin conductance (μS) response over time during negative film exposure for each condition, shown separately for dlPFC (**a**) and vmPFC (**b**) stimulation groups. Skin conductance values reflect difference scores (negative−neutral) across 5 s intervals. Error bars represent ±1 SEM.

A three-way mixed-design ANOVA on SC Δ scores (negative – neutral) showed no main effects of stimulation region, *F*(1, 68) = 1.06, *p* = 0.308, *η*^2^*_p_* = 0.02; time, *F*(5, 340) = 0.21, *p* = 0.959, *η*^2^*_p_* < 0.01; or condition, *F*(3, 204) = 0.88, *p* = 0.455, *η*^2^*_p_* = 0.01. Interactions were also non-significant: Region × Condition, *F*(3, 204) = 0.23, *p* = 0.872, *η*^2^*_p_* < 0.01; Region × Time, *F*(5, 340) = 0.38, *p* = 0.862, *η*^2^*_p_* = 0.01; Condition × Time, *F*(15, 1020) = 0.29, *p* = 0.996, *η*^2^*_p_* < 0.01; Region × Condition × Time, *F*(15, 1020) = 0.98, *p* = 0.470, *η*^2^*_p_* = 0.01. Across tests, effects were small (all *η*^2^*_p_* ≤ 0.02). Accordingly, we found no evidence that SC reactivity was modulated by stimulation site, condition, or time (Figure 7; Table S6).

Planned within-group analyses were likewise null. In the dlPFC group, neither time, *F*(5, 205) = 0.38, *p* = 0.862, *η*^2^*_p_* = 0.01, nor condition, *F*(3, 123) = 0.45, *p* = 0.715, *η*^2^*_p_* = 0.01, nor their interaction, *F*(15, 615) = 0.51, *p* = 0.934, *η*^2^*_p_* = 0.01, reached significance (Figure 7a; see Table S9 for full effect sizes). In the vmPFC group, time, *F*(5, 135) = 0.32, *p* = 0.900, *η*^2^*_p_* = 0.01; condition, *F*(3, 81) = 1.25, *p* = 0.296, *η*^2^*_p_* = 0.04; and the interaction, *F*(15, 405) = 1.20, *p* = 0.273, *η*^2^*_p_* = 0.04, were non-significant (Figure 7b; see Table S10 for full effect sizes).

#### 3.3.3. Respiratory Rate

**Figure 8:**
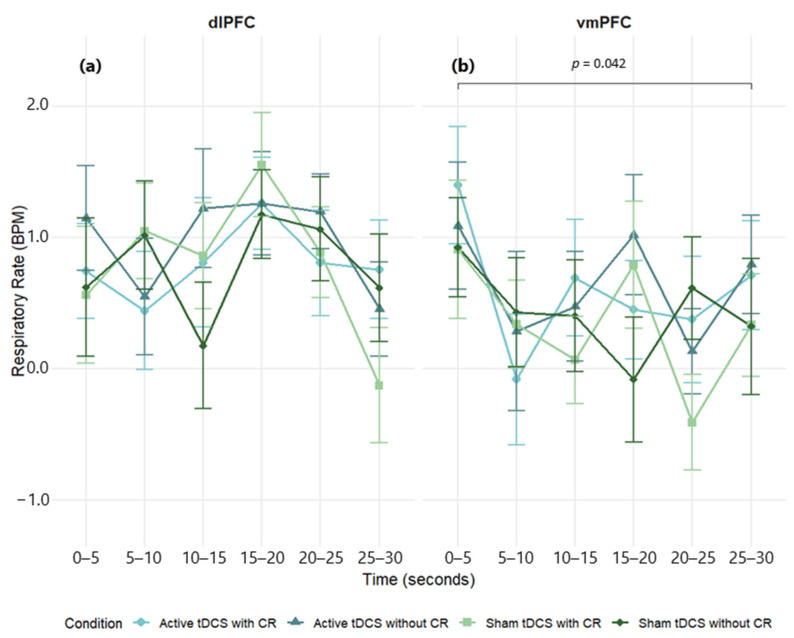
Mean respiratory rate (BPM) over time during exposure to negative film stimuli, presented separately for dlPFC (**a**) and vmPFC (**b**) stimulation groups. Values reflect difference scores (negative−neutral) across 5 s time intervals. Error bars represent ±1 SEM.

A three-way mixed-design ANOVA on respiratory rate (RR) difference scores (negative − neutral) showed a main effect of time, *F*(5, 285) = 2.71, *p* = 0.021, *η*^2^*_p_* = 0.05, but no main effects of condition, *F*(3, 171) = 1.64, *p* = 0.182, *η*^2^*_p_* = 0.03, or region, *F*(5, 57) = 0.55, *p* = 0.461, *η*^2^*_p_* = 0.01. All interactions were non-significant: Region × Condition, *F*(3, 171) = 1.16, *p* = 0.328, *η*^2^*_p_* = 0.02; Region × Time, *F*(5, 285) = 2.16, *p* = 0.059, *η*^2^*_p_* = 0.04; Condition × Time, *F*(15, 855) = 1.08, *p* = 0.372, *η*^2^*_p_* = 0.02; Region × Condition × Time, *F*(15, 855) = 0.75, *p* = 0.734, *η*^2^*_p_* = 0.01. Thus, RR varied modestly over time but was not reliably modulated by stimulation site or condition (Figure 8; Table S6).

Within groups, the dlPFC cohort showed no significant effects of time, *F*(5, 160) = 1.61, *p* = 0.161, *η*^2^*_p_* = 0.05, condition, *F*(3, 96) = 0.31, *p* = 0.815, *η*^2^*_p_* = 0.01, or the condition × time interaction, *F*(15, 480) = 0.91, *p* = 0.551, *η*^2^*_p_* = 0.02 (Figure 8a; see Table S11 for full effect sizes). In the vmPFC cohort, there were main effects of time, *F*(5, 125) = 3.04, *p* = 0.013, *η*^2^*_p_* = 0.11, and condition, *F*(3, 75) = 2.81, *p* = 0.045, *η*^2^*_p_* = 0.10, with a non-significant condition × time interaction, *F*(15, 375) = 0.87, *p* = 0.593, *η*^2^*_p_* = 0.03 (Figure 8b). Post hoc tests for the time factor indicated a reduction at 20–25 s relative to 0–5 s (*p* = 0.042). Given the absent interaction, the condition effect is interpreted cautiously and not localized to specific epochs (see Table S12 for full effect sizes).

### 3.4. Differences in Psychological Activation

#### 3.4.1. Positive and Negative Affect

**Figure 9:**
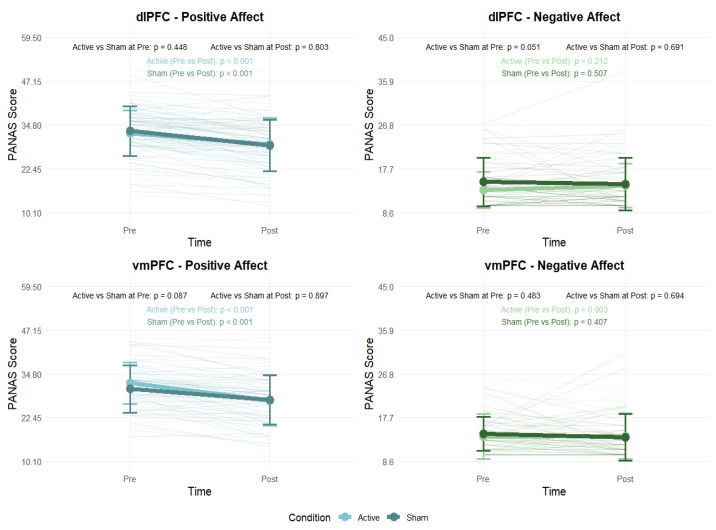
Changes in Positive and Negative Affect as measured by the PANAS before and after stimulation. Error bars indicate ±1 SD.

Paired-samples *t*-tests were conducted to examine changes in positive and negative affect (PANAS) before and after the experimental sessions. In both the dlPFC and vmPFC groups, participants showed a significant decrease in positive affect from pre- to post-session in both the active (dlPFC: *t*(44) = 3.97, *p* < 0.001, *d* = 0.59; vmPFC: *t*(38) = 5.66, *p* < 0.001, *d* = 0.91) and sham (dlPFC: *t*(45) = 7.06, *p* < 0.001, *d* = 1.04; vmPFC: *t*(38) = 4.34, *p* < 0.001, *d* = 0.70) conditions. By contrast, there were no significant changes in negative affect in either the active (*p* > 0.05) or sham (*p* > 0.05) conditions (Figure 9; see Tables S13 and S14).

Additionally, independent-samples *t*-tests were performed to analyze differences in PANAS scores between active and sham conditions at both pre- and post-sessions. The results demonstrated non-significant differences in affect (*p* > 0.05) between the stimulation regions (Figure 9; see Table S15).

#### 3.4.2. tDCS Side Effects

Visual Analogue Scale (VAS) assessments revealed significant differences between pre- and post-session ratings for tDCS side effects. In the dlPFC group, participants receiving active tDCS reported significantly reduced tiredness (*p* < 0.05) and increased itching sensation (*p* < 0.05) following stimulation. Participants in the sham condition reported significantly lower ‘other pain’ ratings post-session (*p* < 0.05) (see Table S16).

Additionally, the sham condition showed a significant increase in reported anxiety (*p* < 0.01) compared to pre-session scores (see Table S17).

#### 3.4.3. Blinding Effectiveness

The blinding questionnaire indicated variability in participants’ awareness of their assigned stimulation condition. In the dlPFC group, approximately 75% of participants correctly identified whether they received active or sham stimulation. In comparison, only about 40% of participants in the vmPFC group correctly identified their condition, suggesting that blinding was more effective for vmPFC stimulation.

## 4. Discussion

This study provides evidence that tDCS targeting the dlPFC enhances ER, particularly when paired with CR. This effect was indexed by significant reductions in HR during the final phase of exposure to emotionally evocative film clips. In contrast, stimulation of the vmPFC produced more variable and statistically nonsignificant effects, which is consistent with its role in affective valuation rather than in top-down regulation. However, despite these trends, the overall analysis did not reveal significant differences between dlPFC and vmPFC stimulation. Although interest in non-invasive brain stimulation for ER is growing, few studies have examined the real-time effects of tDCS in ecologically valid contexts. By combining targeted prefrontal stimulation with naturalistic emotional stimuli and continuous physiological monitoring, this study provides novel evidence that tDCS can modulate autonomic arousal during intentional emotion regulation. These findings offer new insights into the neural and physiological mechanisms of top-down emotion control, with implications for both basic research and clinical neuromodulation strategies.

Despite the late-epoch HR benefit with dlPFC tDCS, we found no reliable modulation of SC or RR and no overall dlPFC–vmPFC difference. Several factors likely contributed. First, a single 20 min/2 mA session may have been sufficient to bias HR but too small to consistently change SC/RR, which are more context- and state-dependent. Second, our 30 s continuous clips with 5 s bins emphasize tonic trends and can dilute phasic SCRs; seated viewing also constrains RR variability. Third, inter-individual differences in autonomic tone and skin/respiratory properties, together with the smaller analytic N for RR, likely reduced sensitivity; effects for SC/RR were uniformly small (η^2^*_p_* ≤ 0.04). Finally, the two-sponge montages used here produce relatively broad current spread, which may have attenuated site-specific contrasts. We therefore interpret the SC/RR nulls cautiously (see Figure 4, Figure 5, Figure 7 and Figure 8; Table S3).

Our findings align with prior research highlighting the role of the dlPFC in sustaining regulatory goals and inhibiting emotional reactivity [10,22]. Specifically, our results indicate that active anodal tDCS over the dlPFC, when paired with CR, resulted in significantly greater decreases in HR compared to both sham stimulation and active tDCS without CR. This finding corroborates the concept that increased excitability of the dlPFC promotes the utilization and execution of top-down regulation techniques, hence enhancing their ability to diminish physiological indicators of emotional arousal [42]. The synergistic interaction between neuromodulation and CR indicates that tDCS may reduce the cognitive burden linked to demanding emotional regulation, potentially by enhancing prefrontal activation related to sustaining regulatory objectives, redirecting attentional focus, or suppressing automatic emotional reactions. The dlPFC has been associated with these processes due to its involvement in working memory, goal maintenance, and the suppression of limbic activity through downstream pathways [13], and our results correspond with this molecular paradigm.

Notably, we showed that active dlPFC stimulation without explicit CR instruction significantly reduced HR, indicating that tDCS alone may produce a broad calming impact on autonomic reactivity. The results may indicate an unmonitored modulation of implicit emotion regulation systems, in which heightened prefrontal excitability amplifies spontaneous regulatory mechanisms absent intentional strategy application. Alternatively, participants may have employed informal or spontaneous regulating techniques during emotional exposure, which were enhanced by the neural priming effects of dlPFC activation. These findings offer converging evidence that dlPFC-targeted tDCS improves both conscious and possibly implicit emotion regulation, as indicated by heart rate, which serves as a sensitive and temporally consistent measure of this modulatory effect.

Emotionally evocative stimuli usually induce a dual-phase autonomic reaction, characterized by an initial deceleration of heart rate indicative of orienting or freezing, succeeded by a subsequent rise as the stimulus is assessed and mobilization ensues [44]. In the current study, it is noteworthy that the application of tDCS over the dlPFC in combination with CR seemed to interfere with or diminish the typical autonomic response pattern. A prolonged decrease in HR that persisted in the final stages of exposure to the stimuli. This pattern suggests that neuromodulation targeting the dlPFC may counteract the natural shift toward sympathetic activation, possibly facilitating sustained top-down regulation and increasing parasympathetic tone [25,45,46], while simultaneously altering threat appraisal and reducing amygdala activity [47].

In the vmPFC group, reductions in HR were noted after active tDCS, regardless of the presence or absence of explicit CR. Nonetheless, these effects exhibited less consistency across timepoints and were more susceptible to statistical correction for multiple comparisons, suggesting that vmPFC stimulation may have a milder or more temporally diffuse impact on autonomic control in comparison to dlPFC stimulation. Although both stimulation sites resulted in decreased heart rate, the effects of dlPFC stimulation were more persistent and pronounced, underscoring its pivotal role in executive control and goal-oriented regulation. The effects of the vmPFC were temporally variable and less dependable, possibly indicating its integrative rather than directing role in emotion processing. This result corresponds with the specific functional role of the vmPFC in emotion regulation, which is believed to be more intricately linked to affective valuation, self-referential processing, and the integration of emotional context, rather than the intentional cognitive control processes generally facilitated by the dlPFC [43,44].

Our vmPFC pattern is consistent with current accounts proposing a dual role for vmPFC in valuation/context integration versus regulatory (safety) modulation. Which function predominates appears state-dependent: under high emotional salience, the vmPFC may prioritize encoding stimulus value and self-relevance, maintaining amygdala responsivity; under lower arousal or learned safety, it more effectively supports inhibitory signaling via vmPFC–amygdala coupling [11,12,13,24]. Given the highly aversive, time-limited clips used here, vmPFC may have been biased toward valuation, yielding attenuated and heterogeneous autonomic effects relative to dlPFC stimulation, which more robustly recruits executive control—especially when paired with reappraisal.

The vmPFC is both physically and functionally linked to limbic and subcortical areas, including the amygdala, hypothalamus, and periaqueductal gray, which are essential for autonomic arousal and threat assessment [45]. The modulation of this network with tDCS may affect emotion processing via implicit or automatic pathways; hence, it impacts arousal control even without the application of conscious strategies. Recent neuroimaging and stimulation research indicates that vmPFC activity facilitates emotion regulation by influencing emotional salience and attenuating amygdala reactivity, frequently via mechanisms that are not readily accessible to introspection or voluntary control [11].

Although a general effect of condition on respiratory activity was observed in the vmPFC group, pairwise comparisons between conditions did not demonstrate statistical significance, suggesting that the modulation may reflect a general temporal trend rather than a condition-specific effect. Consistent with this interpretation, the group showed a significant decrease in respiratory rate during the 20–25 s interval of the film compared to the first 5 s, indicating a progressive modulation of autonomic activity throughout the stimulus. Contrary to expectations, SC and RR exhibited no consistent modulation across circumstances. The findings may indicate modality-specific sensitivities, with heart rate acting as a more temporally responsive and discriminative measure during film-based emotional exposure. Moreover, individual variability and brief clip durations may have attenuated small SC/RR effects. While previous research has indicated a correlation between effective cognitive reappraisal and decreased SCR (e.g., [17]) these effects were not repeated in this study. Multiple factors may explain this gap, such as significant interindividual variability in autonomic responsiveness, the brief duration of film segments, and possible limitations in the sensitivity of SC and RR to identify modest regulatory effects in dynamic, naturalistic tasks. These metrics may be especially susceptible to noise or affected by nonspecific arousal, thereby constraining their discriminative efficacy within the present paradigm. Conversely, HR frequently exhibited considerable variation across experimental. This underscores its role as a more reliable and stable autonomic indicator of regulatory effectiveness in emotionally charged, film-based tasks.

Concerning self-reported affect, both active and sham sessions showed a comparable pre–post decrease in Positive Affect, with no reliable change in Negative Affect. The most likely explanation is a general mood drift driven by sustained exposure to aversive clips, which may mask small, condition-specific effects of a single 20 min tDCS session or brief CR practice; thus, the induction likely exerted a strong main effect on self-report irrespective of stimulation. The absence of PANAS modulation alongside late-epoch HR reductions, therefore, points to a partial dissociation between subjective and autonomic indices of ER. PANAS was sampled only pre–post and aggregates the entire session, whereas the dlPFC-linked autonomic benefit emerged in the final seconds of each clip. Because trial-wise SAM ratings were unavailable due to a technical failure, our subjective measures were likely under-sensitive to brief, time-locked regulation effects. Future studies should align temporal scales by collecting per-clip valence/arousal (or continuous) ratings, including interoceptive/awareness indices, and test longer or repeated stimulation to determine when subjective changes track autonomic modulation.

Although modest, the side effects observed across stimulation conditions may offer meaningful insights into the interpretation of our primary findings. Notably, the reduction in self-reported fatigue following active dlPFC stimulation could be linked to enhanced heart rate modulation, potentially indicating reduced cognitive load as a result of increased dlPFC excitability. While participants consistently reported itching sensations during active stimulation in both prefrontal sites, some site-specific effects also emerged, such as increased anxiety in the sham vmPFC condition, which may reflect the distinct neurofunctional roles and circuit-level connectivity of the dlPFC and vmPFC during emotional processing. Moreover, differences in blinding effectiveness between stimulation sites (75% correct identification for dlPFC vs. 40% for vmPFC) suggest greater detectability of active stimulation in the dlPFC group, which could have introduced expectancy-related influences on the physiological outcomes.

Exploratory analyses of individual differences revealed that participants with greater difficulties in emotion regulation, as assessed by the CERQ, exhibited higher scores not only in maladaptive strategies like rumination and self-blame but also in various adaptive strategies, including acceptance and positive reappraisal. Furthermore, these patients exhibited heightened psychological symptoms across various dimensions. This trend may indicate a compensatory effort to manage discomfort through heightened cognitive exertion. However, it has minimal efficacy. Instead of depending exclusively on maladaptive responses, individuals facing regulatory difficulties seem to employ a broader range of strategies—potentially in an inconsistent or ineffectual manner. These findings support the translational potential of neuromodulation in improving emotion control, especially for those with affective dysregulation. Utilizing ecologically valid stimuli and incorporating physiological and subjective measures, our findings connect controlled cognitive activities. This approach incorporates real-world emotional processing, which enhances both mechanistic comprehension and clinical significance.

Taken together, the dlPFC-linked drop in heart rate late in the clips, especially when participants used reappraisal, points to tDCS + CR as a promising adjunct for conditions with hyperarousal and weak top-down control (e.g., anxiety spectrum, trauma-related disorders) and for mood disorders with reappraisal difficulties. In practice, brief, strategy-linked stimulation could be delivered during exposure or reappraisal exercises, with autonomic markers (HR/HRV, SC) used as near-term targets and montage/polarity tailored to each person’s regulatory profile. Repeated sessions and simple closed-loop timing (e.g., triggering stimulation when arousal rises) may help build and maintain gains. Combining central readouts (e.g., ERPs or amygdala–prefrontal coupling) with peripheral indices would clarify mechanisms and guide individualized protocols.

Several limitations warrant careful consideration. First, the sample was primarily composed of young adult female university students, which restricts the generalizability of findings across broader age, gender, educational, and clinical groups. Future studies should aim for more diverse samples to enhance external validity. Second, although the film paradigm enhances ecological validity, all clips were presented without sound. The absence of prosody, music, and environmental audio likely reduced immersive realism and may have blunted multisensory cues that engage vmPFC networks involved in social/affective appraisal. Future studies should include audio (with standardized levels) and report participants’ subjective engagement. Third, the absence of neuroimaging or electrophysiological data prevents direct inference about the neural mechanisms underlying the observed psycho-physiological changes. Combining tDCS with methods such as fMRI or EEG would improve mechanistic insight. Fourth, while participants were trained in three reappraisal strategies (positive reappraisal, fictional reappraisal, and distancing), these were not analyzed separately. Collapsing across strategies may have masked important differences or site-specific interactions. Fifth, although autonomic measures are informative, SC and RR may have been less sensitive to brief trials or subject to individual variability. Including subjective reports and additional physiological indices (e.g., HRV or pupillometry) could yield a more comprehensive understanding of regulation efficacy. Finally, it is important to note that the observed differences in blinding efficacy across stimulation areas represent a limitation, as expectancy effects may have influenced physiological outcomes, particularly in the dlPFC group, where blinding was less effective.

## 5. Conclusions and Future Directions

This study shows that anodal stimulation of the dlPFC can enhance emotion regulation, especially when combined with cognitive reappraisal strategies. This was reflected in sustained reductions in heart rate, suggesting that dlPFC stimulation may support both deliberate and more automatic regulatory processes. In contrast, vmPFC stimulation produced more variable effects, aligning with its broader role in affective integration rather than direct executive control. The use of emotionally evocative film clips also added ecological validity, bridging controlled experimentation with real-world emotion challenges. Given the small effect sizes and several null findings across modalities, these results should be considered preliminary and warrant replication in larger, preregistered samples using multimodal autonomic indices (e.g., HR/HRV, SC, RR).

Future research should build on these findings by systematically testing how different reappraisal strategies and montage configurations—including unihemispheric, bihemispheric, and interhemispheric approaches—can modulate not only the dlPFC but also broader inhibitory networks relevant for emotion regulation [48]. Combining tDCS with central biomarkers such as ERPs [49] alongside peripheral measures like heart rate and skin conductance could provide a more comprehensive understanding of when and how these networks are engaged.

Importantly, exploring the effects of repetitive stimulation sessions is crucial to determine whether cumulative effects can strengthen and sustain regulatory benefits over time. Recent evidence also shows that individual differences in regulation success and traits are linked to variability in lateral PFC and amygdala activity [50], highlighting the potential for personalized protocols based on neural profiles.

In addition, emerging methods such as tRNS and tACS, especially when frequency- or phase-tuned to individual brain oscillations, deserve further investigation as complementary tools. Altogether, integrating ecologically valid tasks, advanced central and peripheral markers, optimized montage designs, and repeated sessions may substantially advance how neuromodulation protocols are tailored to strengthen emotion regulation in both research and clinical contexts.

Although we focused on autonomic indices, converging evidence suggests that peripheral molecular markers could sharpen mechanistic interpretation in NIBS–emotion-regulation paradigms. Salivary biomarkers indexing arousal/neuromodulatory tone (e.g., orexin-A) have shown sensitivity to non-invasive brain stimulation, circulating microRNAs (miRNAs) are emerging as peripheral readouts of CNS plasticity, and a recent systematic review/meta-analysis indicates that NIBS can increase circulating neurotrophic factors (e.g., BDNF) versus sham [27,28,29]. Future studies should co-record continuous autonomic measures (HR/HRV, SC, RR) with salivary panels and candidate miRNA assays to triangulate mechanisms and move toward individualized neuromodulation protocols.

## Figures and Tables

**Table 1:** Sociodemographic and clinical characteristics.

	dlPFC (*n* = 46)						vmPFC (*n* = 39)								
	*M* (*SD*)/ *n* (%)		*M* (*SD*)/ *n* (%)	*df*	*χ*2/*t*	*p*	*M* (*SD*)/ *n* (%)		*M* (*SD*)/ *n* (%)	*df*	*χ*2/*t*	*p*	*df*	*χ*2/*t*	*p*
	Without ER Difficulties (*n* = 26)	With ER Difficulties (*n* = 20)	dlPFC Total (*n* = 46)				Without ER Difficulties (*n* = 25)	With ER Difficulties (*n* = 14)	vmPFC Total (*n* = 39)						
Age	28.4 (12.6)	28.0 (9.5)	28.2 (11.2)	44	0.13	0.898	27.7 (12.0)	25.8 (6.5)	27.0 (10.3)	37	0.54	0.590	83	0.51	0.613
Sex				1	3.55	0.062				1	2.96	0.123	1	3.46	0.063
Female	22 (84.6)	12 (60.0)	34 (73.9)				24 (96.0)	11 (78.6)	35 (89.7)						
Male	4 (15.4)	8 (40.0)	12 (26.1)				1 (4.0)	3 (21.4)	4 (10.3)						
Nationality				1	1.33	0.435				2	3.50	0.174	2	4.97	0.083
Portuguese	26 (100)	19 (95)	45 (97.8)				23 (92.0)	10 (71.4)	33 (84.6)						
Other nationalities	-	1 (5.0)	1 (2.2)				2 (8.0)	3 (21.4)	5 (12.8)						
Dual nationality	-	-	-				-	1 (7.1)	1 (2.6)						
Marital status				3	5.10	0.164				2	1.86	0.394	3	3.28	0.351
Single	21 (80.8)	15 (75.0)	36 (78.3)				20 (80.0)	13 (92.9)	33 (84.6)						
Cohabiting	1 (3.8)	4 (20.0)	5 (10.9)				2 (8.0)	1 (7.1)	3 (7.7)						
Married	1 (3.8)	1 (5.0)	2 (4.3)				3 (12.0)	-	3 (7.7)						
Divorced	3 (11.5)	-	3 (6.5)				-	-	-						
Education				2	2.25	0.324				2	1.49	0.475	2	4.90	0.086
Middle School	4 (15.4)	1 (5.0)	5 (10.9)				2 (8.0)	-	2 (5.1)						
High School	5 (19.2)	7 (35.0)	12 (26.1)				11 (44.0)	8 (57.1)	19 (48.7)						
Higher Education	17 (65.4)	12 (60.0)	29 (63.0)				12 (48.0)	6 (42.9)	18 (46.2)						
Occupation				4	7.21	0.125				3	3.54	0.316	4	6.02	0.198
Student	13 (50.0)	6 (30.0)	19 (41.3)				15 (60.0)	6 (42.9)	21 (53.8)						
Worker student	1 (3.8)	4 (20.0)	5 (10.9)				3 (12.0)	5 (35.7)	8 (20.5)						
Worker	10 (38.5)	6 (30.0)	16 (34.8)				6 (24.0)	3 (21.4)	9 (23.1)						
Unemployed	1 (3.8)	3 (15.0)	4 (8.7)				1 (4.0)	-	1 (2.6)						
Pensioner or retired	2 (7.7)	-	2 (4.3)				-	-	-						
Alcohol use				1	1.77	0.303				1	0.39	0.609	1	0.06	0.547
Yes	1 (3.8)	3 (15.0)	4 (8.7)				2 (8.0)	2 (14.3)	4 (10.3)						
No	25 (96.2)	17 (85.0)	42 (91.3)				23 (92.0)	12 (85.7)	35 (89.7)						
Tobacco use				1	0.00	0.650				1	0.18	0.595	1	2.27	0.124
Yes	4 (15.4)	3 (15.0)	7 (15.2)				1 (4.0)	13 (92.9)	2 (5.1)						
No	22 (84.6)	17 (85.0)	39 (84.8)				24 (96.0)	1 (7.1)	37 (94.9)						
ER Profile															
Adaptive ER	22.58 (8.98)	27.10 (12.70)	24.54 (10.86)	32.74	−1.35	0.185	16.52 (8.37)	29.64 (10.01)	21.23 (10.92)	37	−4.38	0.000	83	1.40	0.166
Maladaptive ER	3.69 (2.99)	7.30 (4.39)	5.26 (4.05)	31.89	−3.16	0.003	6.68 (5.00)	6.71 (5.68)	6.69 (5.18)	37	−0.02	0.984	83	−1.43	0.156
Cognitive Emotion Regulation															
Acceptance	11.15 (2.87)	14.60 (3.09)	12.65 (3.40)	44	−3.91	0.000	12.04 (3.20)	15.07 (3.32)	13.13 (3.52)	37	−2.81	0.008	83	−0.63	0.528
Positive refocusing	10.96 (2.79)	13.15 (3.63)	11.91 (3.33)	44	−2.31	0.025	10.88 (3.56)	11.50 (4.43)	11.10 (3.85)	37	−0.48	0.636	83	1.04	0.301
Refocus on planning	13.08 (2.70)	15.15 (3.33)	13.98 (3.13)	44	−2.33	0.024	13.16 (3.09)	15.93 (1.56)	14.15 (3.35)	37	−2.67	0.011	83	−0.25	0.803
Positive reappraisal	13.15 (3.55)	14.65 (3.73)	13.80 (3.67)	44	−1.39	0.173	11.96 (3.90)	15.64 (3.88)	13.28 (4.24)	37	−2.84	0.007	83	0.61	0.544
Putting into perspective	11.69 (3.40)	13.50 (3.49)	12.48 (3.52)	44	−1.77	0.084	12.56 (3.53)	12.79 (3.36)	12.64 (3.42)	37	−0.195	0.846	83	−0.22	0.830
Self-blame	8.38 (2.33)	12.15 (2.83)	10.02 (3.16)	44	−4.94	0.000	9.48 (3.31)	12.86 (3.26)	10.69 (3.64)	37	−3.08	0.004	83	−0.91	0.365
Rumination	8.92 (2.21)	15.95 (2.37)	11.98 (4.18)	44	−10.36	0.000	10.40 (1.94)	15.43 (1.56)	12.21 (3.03)	37	−8.32	0.000	81.12	−0.29	0.773
Catastrophizing	6.12 (2.01)	10.25 (3.34)	7.91 (3.35)	29.31	−4.90	0.000	8.00 (2.97)	8.21 (3.62)	8.08 (3.17)	37	−0.20	0.843	83	−0.23	0.819
Blaming others	5.96 (2.24)	10.05 (4.26)	7.74 (3.83)	26.98	−3.90	0.001	6.68 (2.48)	7.93 (3.93)	7.13 (3.09)	37	−1.08	0.296	83	0.80	0.426
Emotional Intelligence	153.27 (19.48)	153.70 (20.10)	153.46 (19.57)	44	−0.07	0.942	150.16 (19.23)	154.43 (24.00)	151.69 (20.85)	37	−0.61	0.547	83	0.40	0.689
Well-being	32.04 (4.70)	34.50 (4.50)	33.11 (4.73)	44	−1.79	0.08	32.00 (6.86)	32.86 (5.83)	32.31 (6.45)	37	−0.39	0.696	68.51	0.64	0.522
Self-control	30.12 (5.07)	26.75 (7.22)	28.65 (6.26)	44	1.86	0.07	28.92 (4.34)	29.43 (5.72)	29.10 (4.81)	37	−0.31	0.756	83	−0.37	0.709
Emotionality	41.92 (6.36)	43.15 (6.90)	42.46 (6.55)	44	−0.63	0.535	40.52 (5.67)	43.00 (6.56)	41.41 (6.04)	37	−1.24	0.223	83	0.76	0.449
Sociability	28.19 (4.83)	28.20 (6.37)	28.20 (5.49)	44	−0.01	0.996	28.00 (6.01)	28.00 (8.26)	28.00 (6.79)	37	0.00	1.00	83	0.15	0.884
Resilience	107.46 (9.29)	107.15 (7.14)	107.33 (8.34)	44	0.12	0.902	105.88 (4.65)	104.50 (5.07)	105.30 (4.78)	37	0.86	0.395	83	1.29	0.202
Family Cohesion	24.46 (2.86)	23.80 (2.55)	24.17 (2.72)	44	0.82	0.419	24.12 (2.16)	22.43 (2.47)	23.51 (2.18)	37	2.48	0.018	83	1.22	0.225
Social Resources	26.31 (4.95)	25.65 (3.05)	26.02 (4.20)	44	0.52	0.604	24.92 (2.16)	25.14 (2.63)	25.00 (2.31)	37	−0.29	0.776	83	1.36	0.179
Social Competence	24.65 (2.94)	24.55 (3.43)	24.61 (3.12)	44	0.11	0.912	23.76 (3.06)	23.57 (3.50)	23.69 (3.18)	37	0.18	0.862	83	1.34	0.185
Planned Future	17.23 (2.03)	17.15 (1.46)	17.20 (1.78)	44	0.15	0.881	17.12 (2.09)	16.57 (2.41)	16.92 (2.19)	37	0.75	0.461	83	0.63	0.529
Perception of Self	22.77 (3.10)	23.10 (3.32)	22.91 (3.17)	44	−0.35	0.730	22.92 (2.48)	23.50 (2.59)	23.13 (2.51)	37	−0.69	0.495	83	−0.34	0.733
Structured Style	16.69 (2.54)	17.45 (2.74)	17.02 (2.63)	44	−0.96	0.338	16.80 (2.63)	16.86 (3.57)	16.92 (2.19)	37	−0.06	0.96	83	0.33	0.741
Mental Illness	36.23 (28.96)	59.15 (37.37)	46.20 (34.46)	44	−2.35	0.024	37.52 (24.23)	53.29 (40.02)	43.18 (31.28)	37	−1.54	0.133	83	0.42	0.676
Somatization	3.42 (4.73)	5.50 (5.58)	4.33 (5.16)	44	−1.37	0.179	2.52 (2.50)	4.00 (4.71)	3.05 (3.47)	37	−1.29	0.206	83	1.31	0.194
Obsession Compulsion	6.81 (3.86)	9.20 (4.26)	7.85 (4.17)	44	−1.99	0.053	7.08 (3.44)	8.29 (3.97)	7.51 (3.63)	37	0.99	0.327	83	0.39	0.697
Interpersonal Sensitivity	2.54 (2.47)	5.40 (4.47)	3.78 (3.73)	44	−2.76	0.008	3.56 (3.23)	4.79 (4.59)	4.00 (3.76)	37	−0.98	0.336	83	−0.27	0.790
Depression	4.46 (4.22)	6.15 (4.69)	5.20 (4.46)	44	−1.28	0.207	3.76 (3.31)	6.43 (6.82)	4.72 (4.95)	16.49	−1.38	0.187	83	0.47	0.641
Anxiety	4.27 (3.75)	7.10 (4.64)	5.50 (4.35)	44	−2.29	0.027	4.64 (2.50)	6.14 (5.88)	5.18 (4.04)	15.67	−0.91	0.376	83	0.35	0.727
Hostility	2.96 (2.79)	5.55 (3.47)	4.09 (3.33)	44	−2.80	0.007	2.96 (2.53)	4.21 (3.62)	3.41 (2.98)	37	−1.27	0.212	83	0.98	0.339
Phobic anxiety	1.88 (3.59)	3.45 (2.80)	2.57 (3.33)	44	−1.61	0.115	2.28 (2.78)	3.00 (3.94)	2.54 (2.21)	37	−0.67	0.509	83	0.04	0.970
Paranoid ideation	4.31 (3.86)	7.40 (4.97)	5.65 (4.59)	44	−2.38	0.022	4.64 (4.10)	5.50 (4.38)	4.95 (4.17)	37	−0.61	0.544	83	0.73	0.465
Psychoticism	2.65 (3.37)	4.65 (4.02)	3.52 (3.76)	44	−1.83	0.074	2.32 (2.46)	5.86 (4.94)	3.59 (3.89)	16.69	−2.51	0.023	83	−0.08	0.935

## Data Availability

The data supporting the findings of this study are not publicly available due to ethical and privacy restrictions imposed by the Ethics Committee for Research in Social and Human Sciences at the University of Minho. However, anonymized summary data may be made available upon reasonable request to the corresponding author or Dr. Catarina Gomes Coelho.

## Supplementary Materials

The following supporting information can be downloaded at: https://www.mdpi.com/article/10.3390/brainsci15090898/s1, Figure S1. Emotion Regulation Training Guidelines; Table S1. Descriptions of the film clips used from the EMDB; Table S2. Physiological Responses (Full clip); Table S3. Heart Rate—Effect Size results two-way ANOVA; Table S4. Skin conductance—effect size results of two-way ANOVA; Table S5. Respiratory rate—effect size results of two-way ANOVA; Table S6. Physiological activation across time (negative − neutral films); Table S7. Heart rate—effect size results of three-way mixed-design ANOVA—dlPFC; Table S8. Heart rate—effect size results of three-way mixed-design ANOVA—vmPFC; Table S9. Skin conductance—effect size results of three-way mixed-design ANOVA—dlPFC; Table S10. skin conductance—effect size results of three-way mixed-design ANOVA—vmPFC; Table S11. Respiratory rate—effect size results of three-way mixed-design ANOVA—dlPFC; Table S12. Respiratory rate—effect size results of three-way mixed-design ANOVA—vmPFC; Table S13. Positive and Negative Affect (PANAS) before and after the experimental sessions—dlPFC; Table S14. PANAS before and after the experimental sessions—vmPFC; Table S15. PANAS between active and sham groups—dlPFC and vmPFC; Table S16. Visual analogue scale for tDCS side effects—dlPFC; Table S17. Visual analogue scale for tDCS side effects—vmPFC. References [6,51,52,53,54,55,56] are cited in the Supplementary Materials.
