# Supplementary material for: From Cortex to Cardiac Response: tDCS of the Prefrontal Cortex Improves Autonomic Markers of Emotion Regulation
Source: Brain Sci. 2025 Aug 22;15(9):898. doi: 10.3390/brainsci15090898 (PMC12467847; doi:10.3390/brainsci15090898)
Supplement: Supplementary file 1 [file brainsci-15-00898-s001.zip › brainsci-3796623-supplementary.pdf]

Supplementary material

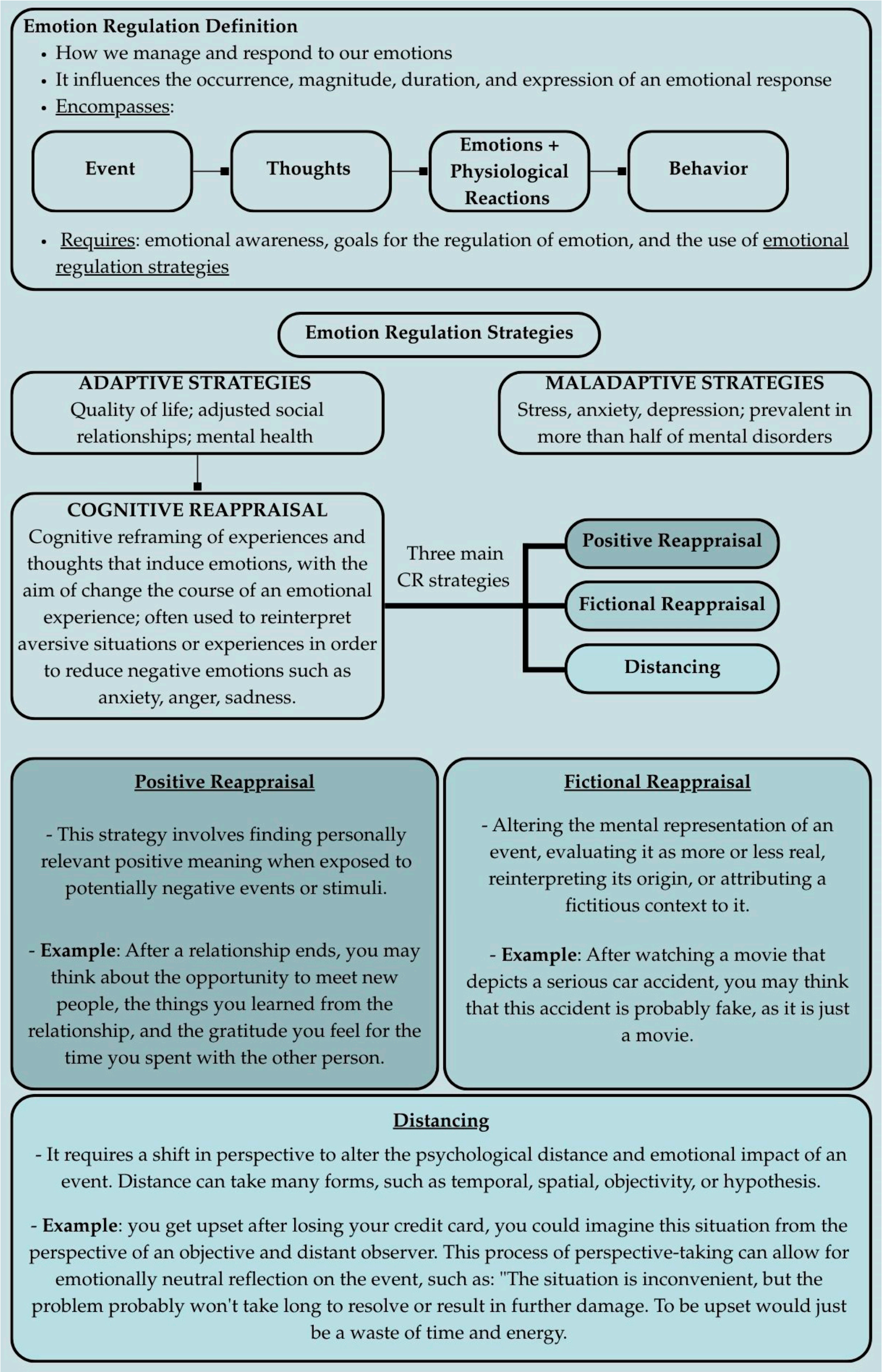

Figure S1. Emotion Regulation Strategies. Overview of adaptive and maladaptive emotion regulation strategies. The diagram illustrates how emotion regulation influences emotional responses and mental health, highlighting cognitive reappraisal and its subtypes (positive reappraisal, fictional reappraisal, and distancing).[1–7].

References

1. Gross, J.J.; Jazaieri, H. Emotion, Emotion Regulation, and Psychopathology: An Affective Science Perspective. *Clinical Psychological Science* 2014, *2*, 387–401, doi:10.1177/2167702614536164.
2. Gross, J.J.; John, O.P. Individual Differences in Two Emotion Regulation Processes: Implications for Affect, Relationships, and Well-Being. *J Pers Soc Psychol* 2003, *85*, 348–362, doi:10.1037/0022-3514.85.2.348.
3. Joormann, J.; Stanton, C.H. Examining Emotion Regulation in Depression: A Review and Future Directions. *Behaviour Research and Therapy* 2016, *86*, 35–49, doi:10.1016/j.brat.2016.07.007.
4. McMahon, T.P.; Naragon-Gainey, K. The Moderating Effect of Maladaptive Emotion Regulation Strategies on Reappraisal: A Daily Diary Study. *Cognit Ther Res* 2018, *42*, 552–564, doi:10.1007/s10608-018-9913-x.
5. Garland, E.; Gaylord, S.; Park, J. The Role of Mindfulness in Positive Reappraisal. *EXPLORE* 2009, *5*, 37–44, doi:10.1016/j.explore.2008.10.001.
6. Makowski, D.; Sperduti, M.; Pelletier, J.; Blondé, P.; La Corte, V.; Arcangeli, M.; Zalla, T.; Lemaire, S.; Dokic, J.; Nicolas, S.; et al. Phenomenal, Bodily and Brain Correlates of Fictional Reappraisal as an Implicit Emotion Regulation Strategy. *Cogn Affect Behav Neurosci* 2019, *19*, 877–897, doi:10.3758/s13415-018-00681-0.
7. Powers, J.P.; LaBar, K.S. Regulating Emotion through Distancing: A Taxonomy, Neurocognitive Model, and Supporting Meta-Analysis. *Neurosci Biobehav Rev* 2019, *96*, 155–173, doi:10.1016/j.neubiorev.2018.04.023.

Table S1. Descriptions of the film clips used from the EMDB.

	**Version A**		**Valence** M(SD)	**Arousal** M(SD)	**Version B**		**Valence** M(SD)	**Arousal** M(SD)
**Negative**	**1,001**	Leatherface removing the face of Mathew Bomer	1.68 (1.45)	7.45 (1.77)	**1,002**	Vinnie Jones removing the eyes and the teeth of the victim	1.67 (1.36)	7.72 (1.67)
	**1,009**	Vinnie Jones with a vicious attack direct to a woman that ends with the decapitation of the victim	1.83 (1.24)	6.88 (1.70)	**1,007**	Jordana Brewster very scared, hidden on a box assists to a mutilation of her boyfriend	1.81 (1.43)	7.33 (1.91)
	**7,000**	Teenage boy abused by a group of young people on the street	1.92 (1.15)	6.21 (2.23)	**1,008**	Jaimie Alexander giving a merciful shot to the head of a police	1.94 (1.41)	6.53 (2.05)
	**1,006**	Savage attack from cannibal tribe to one anthropologist, dismembering him. Children start to eat parts of him	1.98 (1.50)	7.37 (1.88)	**7,001**	Teenage boy attacked by a group of young people in the toilet at the school	1.95 (1.37)	6.48 (2.30)
	**1,000**	Amputation scene on top of the ruins	2.04 (1.98)	7.11 (1.77)	**7,009**	Homeless humiliated on the street	2.00 (1.29)	6.31 (2.29)
	**7,007**	Young woman filmed while being assaulted in a house	2.05 (1.27)	6.13 (2.33)	**1,005**	Leslie Bibb inside a carriage with bodies hanging from the ceiling	2.06 (1.48)	6.92 (1.74)
	**7,002**	Teenage girl beaten up in the classroom by other classmates	2.18 (1.27)	5.94 (2.38)	**1,004**	Cannibalism scene	2.07 (1.91)	6.88 (1.95)
	**7,003**	Teenage boy humiliated in locker room	2.18 (1.27)	5.85 (2.43)	**7,006**	Teenage boy assaulted in the school hallway by a group of students	2.25 (1.44)	5.54 (2.21)
	**7,005**	Schoolmates bully a little boy	2.29 (1.39)	5.80 (2.37)	**7,004**	Little boy attacked by a group of older children and dragged into the dustbin on the street	2.29 (1.32)	5.79 (2.35)
	**3,006**	Josh Hutcherson crying in the arms of Robert Patrick	2.88 (1.36)	4.25 (2.12)	**7,008**	Teenage boy attacked in the sports court	2.37 (1.48)	5.70 (2.25)

	**1,003**	Jay Hernandez is being tortured on a chair and fingers form his hand are amputated	2.99 (2.00)	6.19 (2.20)	**3,003**	Very sad Belen Fabra considering suicide	2.65 (1.42)	4.29 (2.02)
	**3,005**	Danielle Savre crying in the arms of Matt Cohen	3.54 (1.53)	4.05 (1.96)	**3,001**	Girl crying while her beloved died in her arms	3.04 (1.67)	4.79 (1.42)
**Neutral**	**6,009**	Moving small, coloured paper triangles	4.98 (0.73)	2.14 (1.68)	**6,010**	Handling a small wooden puppet	5.06 (0.91)	2.20 (1.73)
	**6,013**	Moving coloured paper trapezoids	4.91 (0.94)	2.21 (1.97)	**6,011**	Gait in different shoes/sleepers	5.08 (1.02)	2.50 (1.84)
	**6,004**	Moving coloured paper squares	4.90 (0.94)	2.01 (1.82)	**6,005**	Moving coloured cups	5.11 (1.00)	2.15 (1.84)
	**6,012**	Putting cutlery in a tray	5.13 (0.65)	1.97 (1.60)	**6,003**	Moving highlighters of different colours	4.89 (1.09)	2.39 (2.04)
	**6,002**	Moving big, coloured paper triangles	5.14 (0.75)	1.85 (1.51)	**6,008**	Bouncing ball slowly moving in the hall	5.19 (0.95)	2.15 (1.87)
	**6,006**	Painting a leaf	5.33 (1.32)	2,66 (1.92)	**6,007**	Solving three different mazes	5.19 (1.34)	2,63 (2.07)

Table S2. Physiological Responses (Full clip)

	Brain Region	Condition	*M*	*SD*	SEM
Heart Rate	dlPFC	Active tDCS with CR	74.68	10.53	1.55
		Active tDCS without CR	74.92	10.97	1.62
		Sham tDCS with CR	76.97	9.83	1.45
		Sham tDCS without CR	77.49	10.38	1.53
	vmPFC	Active tDCS with CR	73.27	11.72	1.88
		Active tDCS without CR	73.17	11.83	1.89
		Sham tDCS with CR	74.87	12.84	2.06
		Sham tDCS without CR	75.19	12.81	2.05
Skin Conductance	dlPFC	Active tDCS with CR	-.01	.12	.02
		Active tDCS without CR	-.03	.12	.02
		Sham tDCS with CR	-.002	.11	.02
		Sham tDCS without CR	-.02	.06	.01
	vmPFC	Active tDCS with CR	-.01	.10	.02
		Active tDCS without CR	-.03	.07	.01
		Sham tDCS with CR	.01	.09	.02
		Sham tDCS without CR	.01	.11	.02
Respiratory Rate	dlPFC	Active tDCS with CR	17.02	2.44	.36
		Active tDCS without CR	17.14	2.59	.38
		Sham tDCS with CR	17.23	2.70	.40
		Sham tDCS without CR	17.19	2.56	.34
	vmPFC	Active tDCS with CR	17.57	2.43	.39
		Active tDCS without CR	17.82	2.61	.42
		Sham tDCS with CR	17.13	3.13	.51
		Sham tDCS without CR	17.36	3.13	.50

Table S3. Heart Rate - Effect Size results two-way ANOVA

Condition	Cohen's *d*	Condition - dlPFC	Cohen's *d*	Condition - vmPFC	Cohen's *d*	Group	Condition	Cohen's *d*
Ativa+CR vs Ativa-CR	.008	Ativa+CR vs Ativa-CR	.022	Ativa+CR vs Ativa-CR	.008	dlPFC vs vmPFC	Ativa+CR	.127
Ativa+CR vs Sham+CR	.177	Ativa+CR vs Sham+CR	.225	Ativa+CR vs Sham+CR	.131		Ativa-CR	.154
Ativa+CR vs Sham-CR	.212*	Ativa+CR vs Sham-CR	.269	Ativa+CR vs Sham-CR	.156		Sham+CR	.186
Ativa-CR vs Sham+CR	.167	Ativa-CR vs Sham+CR	.197	Ativa-CR vs Sham+CR	.138		Sham-CR	.199
Ativa-CR vs Sham-CR	.202*	Ativa-CR vs Sham-CR	.240	Ativa-CR vs Sham-CR	.163			
Sham+CR vs Sham-CR	.037	Sham+CR vs Sham-CR	.051	Sham+CR vs Sham-CR	.024			

Note. Ativa+CR = active condition with cognitive reappraisal; Ativa-CR = active condition without cognitive reappraisal; Sham+CR = sham condition with cognitive reappraisal; Sham-CR = sham condition without cognitive reappraisal

Table S4. Skin conductance - effect size results of two-way ANOVA

Condition	Cohen's *d*	Condition - dlPFC	Cohen's *d*	Condition - vmPFC	Cohen's *d*	Group	Condition	Cohen's *d*
Ativa+CR vs Ativa-CR	-.438	Ativa+CR vs Ativa-CR	-.436	Ativa+CR vs Ativa-CR	.416	dlPFC vs vmPFC	Ativa+CR	.414
Ativa+CR vs Sham+CR	.336	Ativa+CR vs Sham+CR	.314	Ativa+CR vs Sham+CR	-.269		Ativa-CR	-.254
Ativa+CR vs Sham-CR	.237	Ativa+CR vs Sham-CR	-.226	Ativa+CR vs Sham-CR	-.205		Sham+CR	-.198
Ativa-CR vs Sham+CR	.191	Ativa-CR vs Sham+CR	.168	Ativa-CR vs Sham+CR	.164		Sham-CR	.155
Ativa-CR vs Sham-CR	.150	Ativa-CR vs Sham-CR	.143	Ativa-CR vs Sham-CR	.127			
Sham+CR vs Sham-CR	.115	Sham+CR vs Sham-CR	-.104	Sham+CR vs Sham-CR	.102			

Note. Ativa+CR = active condition with cognitive reappraisal; Ativa-CR = active condition without cognitive reappraisal; Sham+CR = sham condition with cognitive reappraisal; Sham-CR = sham condition without cognitive reappraisal

Table S5. Respiratory rate - effect size results of two-way ANOVA

Condition	Cohen's *d*	Condition - dlPFC	Cohen's *d*	Condition - vmPFC	Cohen's *d*	Group	Condition	Cohen's *d*
Ativa+CR vs Ativa-CR	.069	Ativa+CR vs Ativa-CR	-.047	Ativa+CR vs Ativa-CR	-.099	dlPFC vs vmPFC	Ativa+CR	.127
Ativa+CR vs Sham+CR	.031	Ativa+CR vs Sham+CR	-.082	Ativa+CR vs Sham+CR	.154		Ativa-CR	.154
Ativa+CR vs Sham-CR	.000	Ativa+CR vs Sham-CR	-.074	Ativa+CR vs Sham-CR	.075		Sham+CR	.186
Ativa-CR vs Sham+CR	.094	Ativa-CR vs Sham+CR	-.035	Ativa-CR vs Sham+CR	.236		Sham-CR	.353
Ativa-CR vs Sham-CR	.066	Ativa-CR vs Sham-CR	-.023	Ativa-CR vs Sham-CR	.159			
Sham+CR vs Sham-CR	-.030	Sham+CR vs Sham-CR	.014	Sham+CR vs Sham-CR	-.071			

Note. Ativa+CR = active condition with cognitive reappraisal; Ativa-CR = active condition without cognitive reappraisal; Sham+CR = sham condition with cognitive reappraisal; Sham-CR = sham condition without cognitive reappraisal

Table S6. Physiological activation across time (negative – neutral films)

	Brain Region	Time	Condition	*M*	*SD*	SEM
Heart Rate	dlPFC	0-5 seconds	Active tDCS with CR	.86	4.93	.73
			Active tDCS without CR	1.15	4.26	.63
			Sham tDCS with CR	.25	3.44	.51
			Sham tDCS without CR	.08	3.57	.53
	vmPFC	0-5 seconds	Active tDCS with CR	-.06	4.84	.76
			Active tDCS without CR	-.54	3.87	.62
			Sham tDCS with CR	-.12	4.36	.70
			Sham tDCS without CR	-.53	4.86	.78
	dlPFC	5-10 seconds	Active tDCS with CR	.03	4.59	.68
			Active tDCS without CR	.26	3.24	.48
			Sham tDCS with CR	.91	4.15	.61
			Sham tDCS without CR	.54	3.51	.52
	vmPFC	5-10 seconds	Active tDCS with CR	.06	4.21	.67
			Active tDCS without CR	-1.09	3.60	.58
			Sham tDCS with CR	-.77	5.36	.86
			Sham tDCS without CR	-.40	4.26	.68
	dlPFC	10-15 seconds	Active tDCS with CR	.00	3.74	.55
			Active tDCS without CR	-.31	3.04	.45
			Sham tDCS with CR	-1.41	2.88	.42
			Sham tDCS without CR	-.54	3.30	.49
	vmPFC	10-15 seconds	Active tDCS with CR	-.80	4.65	.74
			Active tDCS without CR	-1.12	4.06	.65
			Sham tDCS with CR	-.95	3.73	.60
			Sham tDCS without CR	-.38	3.51	.56
	dlPFC	15-20 seconds	Active tDCS with CR	-.04	3.79	.56
			Active tDCS without CR	.22	3.04	.45
			Sham tDCS with CR	-.91	3.83	.56
			Sham tDCS without CR	-.21	3.22	.48
	vmPFC	15-20 seconds	Active tDCS with CR	-.19	2.86	.46
			Active tDCS without CR	-.10	3.67	.59
			Sham tDCS with CR	-.05	2.90	.47
			Sham tDCS without CR	-.40	2.61	.42
	dlPFC	20-25 seconds	Active tDCS with CR	-.12	3.31	.49
			Active tDCS without CR	-.17	3.15	.46
			Sham tDCS with CR	-1.23	4.11	.61
			Sham tDCS without CR	.10	3.31	.49
	vmPFC	20-25 seconds	Active tDCS with CR	-1.58	3.78	.61
			Active tDCS without CR	-1.42	3.26	.52

			Sham tDCS with CR	-1.03	3.46	.55
			Sham tDCS without CR	-.77	2.98	.48
	dlPFC	25-30 seconds	Active tDCS with CR	-1.15	3.37	.50
			Active tDCS without CR	-.33	2.82	.42
			Sham tDCS with CR	-.74	3.79	.56
			Sham tDCS without CR	.21	3.18	.47
	vmPFC	25-30 seconds	Active tDCS with CR	-1.88	3.14	.50
			Active tDCS without CR	-.74	3.27	.52
			Sham tDCS with CR	-1.66	4.29	.69
			Sham tDCS without CR	-.32	3.10	.50
Skin Conductance	dlPFC	0-5 seconds	Active tDCS with CR	.03	.53	.08
			Active tDCS without CR	.05	.96	.14
			Sham tDCS with CR	.02	.71	.11
			Sham tDCS without CR	.04	.81	.12
	vmPFC	0-5 seconds	Active tDCS with CR	-.00	.22	.04
			Active tDCS without CR	-.10	.41	.07
			Sham tDCS with CR	.10	.51	.09
			Sham tDCS without CR	.17	.57	.10
	dlPFC	5-10 seconds	Active tDCS with CR	.07	.57	.08
			Active tDCS without CR	.04	.35	.05
			Sham tDCS with CR	-.08	.53	.08
			Sham tDCS without CR	-.05	.25	.04
	vmPFC	5-10 seconds	Active tDCS with CR	-.01	.25	.04
			Active tDCS without CR	.01	.31	.05
			Sham tDCS with CR	.01	.41	.07
			Sham tDCS without CR	.01	.40	.07
	dlPFC	10-15 seconds	Active tDCS with CR	-.01	.40	.06
			Active tDCS without CR	-.07	.40	.06
			Sham tDCS with CR	.09	.65	.10
			Sham tDCS without CR	.05	.33	.05
	vmPFC	10-15 seconds	Active tDCS with CR	.09	.42	.07
			Active tDCS without CR	.11	.39	.07
			Sham tDCS with CR	-.04	.37	.06
			Sham tDCS without CR	-.05	.53	.09
	dlPFC	15-20 seconds	Active tDCS with CR	-.09	.40	.06
			Active tDCS without CR	-.03	.31	.05
			Sham tDCS with CR	.03	.36	.05
			Sham tDCS without CR	-.01	.40	.06
	vmPFC	15-20 seconds	Active tDCS with CR	.07	.16	.03
			Active tDCS without CR	.00	.16	.03
			Sham tDCS with CR	-.03	.57	.10

			Sham tDCS without CR	.07	.41	.07
	dlPFC	20-25 seconds	Active tDCS with CR	.05	.47	.07
			Active tDCS without CR	-.03	.36	.05
			Sham tDCS with CR	.06	.51	.08
			Sham tDCS without CR	-.02	.46	.07
	vmPFC	20-25 seconds	Active tDCS with CR	-.04	.29	.05
			Active tDCS without CR	-.01	.32	.06
			Sham tDCS with CR	.12	.40	.07
			Sham tDCS without CR	-.01	.33	.06
	dlPFC	25-30 seconds	Active tDCS with CR	-.01	.36	.05
			Active tDCS without CR	-.06	.52	.08
			Sham tDCS with CR	-.03	.29	.04
			Sham tDCS without CR	-.11	.41	.06
	vmPFC	25-30 seconds	Active tDCS with CR	-.03	.27	.05
			Active tDCS without CR	.01	.28	.05
			Sham tDCS with CR	-.04	.37	.06
			Sham tDCS without CR	.06	.44	.07
Respiratory Rate	dlPFC	0-5 seconds	Active tDCS with CR	.75	2.45	.36
			Active tDCS without CR	1.47	2.68	.40
			Sham tDCS with CR	.56	3.43	.52
			Sham tDCS without CR	.62	3.43	.53
	vmPFC	0-5 seconds	Active tDCS with CR	1.40	2.68	.45
			Active tDCS without CR	1.09	2.94	.43
			Sham tDCS with CR	.91	3.15	.52
			Sham tDCS without CR	.93	2.25	.38
	dlPFC	5-10 seconds	Active tDCS with CR	.44	2.99	.45
			Active tDCS without CR	.55	2.94	.44
			Sham tDCS with CR	1.05	2.38	.36
			Sham tDCS without CR	1.02	2.65	.41
	vmPFC	5-10 seconds	Active tDCS with CR	-.08	2.98	.50
			Active tDCS without CR	.29	3.54	.61
			Sham tDCS with CR	.34	2.02	.34
			Sham tDCS without CR	.43	2.52	.42
	dlPFC	10-15 seconds	Active tDCS with CR	.81	3.33	.49
			Active tDCS without CR	1.22	3.06	.45
			Sham tDCS with CR	.86	2.66	.41
			Sham tDCS without CR	.18	3.15	.48
	vmPFC	10-15 seconds	Active tDCS with CR	.69	2.62	.44
			Active tDCS without CR	.47	4.47	.42
			Sham tDCS with CR	.07	2.03	.33
			Sham tDCS without CR	.40	2.60	.43

	dlPFC	15-20 seconds	Active tDCS with CR	1.26	2.36	.35
			Active tDCS without CR	1.26	2.61	.39
			Sham tDCS with CR	1.56	2.57	.40
			Sham tDCS without CR	1.18	2.16	.34
	vmPFC	15-20 seconds	Active tDCS with CR	.45	2.22	.38
			Active tDCS without CR	1.02	2.78	.46
			Sham tDCS with CR	.79	2.96	.49
			Sham tDCS without CR	-.08	2.89	.48
	dlPFC	20-25 seconds	Active tDCS with CR	.81	2.64	.40
			Active tDCS without CR	1.20	1.87	.29
			Sham tDCS with CR	.89	2.24	.35
			Sham tDCS without CR	1.07	2.57	.40
	vmPFC	20-25 seconds	Active tDCS with CR	.38	2.96	.38
			Active tDCS without CR	.13	1.95	.33
			Sham tDCS with CR	-.41	2.15	.36
			Sham tDCS without CR	.62	2.32	.39
	dlPFC	25-30 seconds	Active tDCS with CR	.76	2.53	.37
			Active tDCS without CR	.46	2.43	.36
			Sham tDCS with CR	-.13	2.90	.44
			Sham tDCS without CR	.62	2.65	.41
	vmPFC	25-30 seconds	Active tDCS with CR	.71	2.48	.41
			Active tDCS without CR	.80	2.27	.37
			Sham tDCS with CR	.33	2.31	.39
			Sham tDCS without CR	.32	3.14	.52

Table S7. Heart rate - effect size results of three-way mixed-design ANOVA - dlPFC

				Condition x Time		
Condition	Cohen's *d*	Time	Cohen's *d*	Interval	Condition	Cohen's *d*
Ativa+CR vs Ativa-CR	.076	0-5 vs 5-10	.038	0-5 s	Ativa+CR vs Ativa-CR	.062
Ativa+CR vs Sham+CR	.095	0-5 vs 10-15	.304*		Ativa+CR vs Sham+CR	.143
Ativa+CR vs Sham-CR	.048	0-5 vs 15-20	.216		Ativa+CR vs Sham-CR	.182
Ativa-CR vs Sham+CR	.182	0-5 vs 20-25	.247		Ativa-CR vs Sham+CR	.231
Ativa-CR vs Sham-CR	.031	0-5 vs 25-30	.324*		Ativa-CR vs Sham-CR	.272
Sham+CR vs Sham-CR	.154	5-10 vs 10-15	.272		Sham+CR vs Sham-CR	.049
		5-10 vs 15-20	.181	5-10 s	Ativa+CR vs Ativa-CR	.057
		5-10 vs 20-25	.214		Ativa+CR vs Sham+CR	.202
		5-10 vs 25-30	.292		Ativa+CR vs Sham-CR	.125
		10-15 vs 15-20	.096		Ativa-CR vs Sham+CR	.176
		10-15 vs 20-25	.061		Ativa-CR vs Sham-CR	.083
		10-15 vs 25-30	.017		Sham+CR vs Sham-CR	.098
		15-20 vs 20-25	.035	10-15 s	Ativa+CR vs Ativa-CR	.083
		15-20 vs 25-30	.114		Ativa+CR vs Sham+CR	.423
		20-25 vs 25-30	.078		Ativa+CR vs Sham-CR	.154
					Ativa-CR vs Sham+CR	.324
					Ativa-CR vs Sham-CR	.064
					Sham+CR vs Sham-CR	.281
				15-20 s	Ativa+CR vs Ativa-CR	.075
					Ativa+CR vs Sham+CR	.229
					Ativa+CR vs Sham-CR	.050
					Ativa-CR vs Sham+CR	.326
					Ativa-CR vs Sham-CR	.137
					Sham+CR vs Sham-CR	.197
				20-25 s	Ativa+CR vs Ativa-CR	.016
					Ativa+CR vs Sham+CR	.299
					Ativa+CR vs Sham-CR	.065
					Ativa-CR vs Sham+CR	.291
					Ativa-CR vs Sham-CR	.083
					Sham+CR vs Sham-CR	.357
				25-30 s	Ativa+CR vs Ativa-CR	.421
					Ativa+CR vs Sham+CR	.252
					Ativa+CR vs Sham-CR	.565*
					Ativa-CR vs Sham+CR	.122
					Ativa-CR vs Sham-CR	.180
					Sham+CR vs Sham-CR	.272

Note. Ativa+CR = active condition with cognitive reappraisal; Ativa-CR = active condition without cognitive reappraisal; Sham+CR = sham condition with cognitive reappraisal; Sham-CR = sham condition without cognitive reappraisal

Table S8. Heart rate - effect size results of three-way mixed-design ANOVA - vmPFC

				Condition x Time		
Condition	Cohen's *d*	Time	Cohen's *d*	Interval	Condition	Cohen's *d*
Ativa+CR vs Ativa-CR	.024	0-5 vs 5-10	.055	0-5 s	Ativa+CR vs Ativa-CR	.110
Ativa+CR vs Sham+CR	.005	0-5 vs 10-15	.119		Ativa+CR vs Sham+CR	.013
Ativa+CR vs Sham-CR	.072	0-5 vs 15-20	.033		Ativa+CR vs Sham-CR	.098
Ativa-CR vs Sham+CR	.019	0-5 vs 20-25	.225		Ativa-CR vs Sham+CR	.102
Ativa-CR vs Sham-CR	.102	0-5 vs 25-30	.209		Ativa-CR vs Sham-CR	.002
Sham+CR vs Sham-CR	.077	5-10 vs 10-15	.063		Sham+CR vs Sham-CR	.090
		5-10 vs 15-20	.097	5-10 s	Ativa+CR vs Ativa-CR	.294
		5-10 vs 20-25	.166		Ativa+CR vs Sham+CR	.172
		5-10 vs 25-30	.150		Ativa+CR vs Sham-CR	.109
		10-15 vs 15-20	.178		Ativa-CR vs Sham+CR	.071
		10-15 vs 20-25	.104		Ativa-CR vs Sham-CR	.175
		10-15 vs 25-30	.089		Sham+CR vs Sham-CR	.076
		15-20 vs 20-25	.317	10-15 s	Ativa+CR vs Ativa-CR	.072
		15-20 vs 25-30	.294		Ativa+CR vs Sham+CR	.034
		20-25 vs 25-30	.015		Ativa+CR vs Sham-CR	.102
					Ativa-CR vs Sham+CR	.044
					Ativa-CR vs Sham-CR	.194
					Sham+CR vs Sham-CR	.156
				15-20 s	Ativa+CR vs Ativa-CR	.029
					Ativa+CR vs Sham+CR	.050
					Ativa+CR vs Sham-CR	.075
					Ativa-CR vs Sham+CR	.014
					Ativa-CR vs Sham-CR	.094
					Sham+CR vs Sham-CR	.126
				20-25 s	Ativa+CR vs Ativa-CR	.043
					Ativa+CR vs Sham+CR	.151
					Ativa+CR vs Sham-CR	.238
					Ativa-CR vs Sham+CR	.117
					Ativa-CR vs Sham-CR	.211
					Sham+CR vs Sham-CR	.082
				25-30 s	Ativa+CR vs Ativa-CR	.355
					Ativa+CR vs Sham+CR	.057
					Ativa+CR vs Sham-CR	.501
					Ativa-CR vs Sham+CR	.242
					Ativa-CR vs Sham-CR	.133
					Sham+CR vs Sham-CR	.359

Note. Ativa+CR = active condition with cognitive reappraisal; Ativa-CR = active condition without cognitive reappraisal; Sham+CR = sham condition with cognitive reappraisal; Sham-CR = sham condition without cognitive reappraisal

Table S9. Skin conductance - effect size results of three-way mixed-design ANOVA - dlPFC

Condition	Cohen's *d*	Time	Cohen's *d*	Condition x Time: Interval	Condition x Time: Condition	Condition x Time: Cohen's *d*
Ativa+CR vs Ativa-CR	.041	0-5 vs 5-10	.066	0-5 s	Ativa+CR vs Ativa-CR	.023
Ativa+CR vs Sham+CR	.024	0-5 vs 10-15	.039		Ativa+CR vs Sham+CR	.013
Ativa+CR vs Sham-CR	.045	0-5 vs 15-20	.107		Ativa+CR vs Sham-CR	.016
Ativa-CR vs Sham+CR	.060	0-5 vs 20-25	.035		Ativa-CR vs Sham+CR	.031
Ativa-CR vs Sham-CR	.001	0-5 vs 25-30	.148		Ativa-CR vs Sham-CR	.007
Sham+CR vs Sham-CR	.065	5-10 vs 10-15	.036		Sham+CR vs Sham-CR	.026
		5-10 vs 15-20	.056	5-10 s	Ativa+CR vs Ativa-CR	.059
		5-10 vs 20-25	.043		Ativa+CR vs Sham+CR	.265
		5-10 vs 25-30	.115		Ativa+CR vs Sham-CR	.254
		10-15 vs 15-20	.095		Ativa-CR vs Sham+CR	.266
		10-15 vs 20-25	.007		Ativa-CR vs Sham-CR	.282
		10-15 vs 25-30	.151		Sham+CR vs Sham-CR	.079
		15-20 vs 20-25	.103	10-15 s	Ativa+CR vs Ativa-CR	.150
		15-20 vs 25-30	.067		Ativa+CR vs Sham+CR	.187
		20-25 vs 25-30	.159		Ativa+CR vs Sham-CR	.166
					Ativa-CR vs Sham+CR	.299
					Ativa-CR vs Sham-CR	.331
					Sham+CR vs Sham-CR	.075
				15-20 s	Ativa+CR vs Ativa-CR	.186
					Ativa+CR vs Sham+CR	.328
					Ativa+CR vs Sham-CR	.207
					Ativa-CR vs Sham+CR	.174
					Ativa-CR vs Sham-CR	.046
					Sham+CR vs Sham-CR	.110
				20-25 s	Ativa+CR vs Ativa-CR	.178
					Ativa+CR vs Sham+CR	.032
					Ativa+CR vs Sham-CR	.144
					Ativa-CR vs Sham+CR	.203
					Ativa-CR vs Sham-CR	.017
					Sham+CR vs Sham-CR	.169
				25-30 s	Ativa+CR vs Ativa-CR	.102
					Ativa+CR vs Sham+CR	.053
					Ativa+CR vs Sham-CR	.263
					Ativa-CR vs Sham+CR	.067
					Ativa-CR vs Sham-CR	.117
					Sham+CR vs Sham-CR	.235

Note. Ativa+CR = active condition with cognitive reappraisal; Ativa-CR = active condition without cognitive reappraisal; Sham+CR = sham condition with cognitive reappraisal; Sham-CR = sham condition without cognitive reappraisal

Table S10. skin conductance - effect size results of three-way mixed-design ANOVA - vmPFC

				Condition x Time		
Condition	Cohen's *d*	Time	Cohen's *d*	Interval	Condition	Cohen's *d*
Ativa+CR vs Ativa-CR	.033	0-5 vs 5-10	.092	0-5 s	Ativa+CR vs Ativa-CR	.300
Ativa+CR vs Sham+CR	.021	0-5 vs 10-15	.042		Ativa+CR vs Sham+CR	.260
Ativa+CR vs Sham-CR	.080	0-5 vs 15-20	.036		Ativa+CR vs Sham-CR	.395
Ativa-CR vs Sham+CR	.045	0-5 vs 20-25	.069		Ativa-CR vs Sham+CR	.434
Ativa-CR vs Sham-CR	.102	0-5 vs 25-30	.106		Ativa-CR vs Sham-CR	.542
Sham+CR vs Sham-CR	.051	5-10 vs 10-15	.047		Sham+CR vs Sham-CR	.130
		5-10 vs 15-20	.062	5-10 s	Ativa+CR vs Ativa-CR	.077
		5-10 vs 20-25	.027		Ativa+CR vs Sham+CR	.065
		5-10 vs 25-30	.017		Ativa+CR vs Sham-CR	.077
		10-15 vs 15-20	.009		Ativa-CR vs Sham+CR	.000
		10-15 vs 20-25	.024		Ativa-CR vs Sham-CR	.011
		10-15 vs 25-30	.062		Sham+CR vs Sham-CR	.010
		15-20 vs 20-25	.036	10-15 s	Ativa+CR vs Ativa-CR	.054
		15-20 vs 25-30	.079		Ativa+CR vs Sham+CR	.337
		20-25 vs 25-30	.044		Ativa+CR vs Sham-CR	.288
					Ativa-CR vs Sham+CR	.404
					Ativa-CR vs Sham-CR	.341
					Sham+CR vs Sham-CR	.006
				15-20 s	Ativa+CR vs Ativa-CR	.436
					Ativa+CR vs Sham+CR	.228
					Ativa+CR vs Sham-CR	.013
					Ativa-CR vs Sham+CR	.063
					Ativa-CR vs Sham-CR	.210
					Sham+CR vs Sham-CR	.184
				20-25 s	Ativa+CR vs Ativa-CR	.098
					Ativa+CR vs Sham+CR	.461
					Ativa+CR vs Sham-CR	.119
					Ativa-CR vs Sham+CR	.362
					Ativa-CR vs Sham-CR	.023
					Sham+CR vs Sham-CR	.336
				25-30 s	Ativa+CR vs Ativa-CR	.143
					Ativa+CR vs Sham+CR	.017
					Ativa+CR vs Sham-CR	.258
					Ativa-CR vs Sham+CR	.136
					Ativa-CR vs Sham-CR	.149
					Sham+CR vs Sham-CR	.247

Note. Ativa+CR = active condition with cognitive reappraisal; Ativa-CR = active condition without cognitive reappraisal; Sham+CR = sham condition with cognitive reappraisal; Sham-CR = sham condition without cognitive reappraisal

Table S11. Respiratory rate - effect size results of three-way mixed-design ANOVA - dlPFC

				Condition x Time		
Condition	Cohen's *d*	Time	Cohen's *d*	Interval	Condition	Cohen's *d*
Ativa+CR vs Ativa-CR	.062	0-5 vs 5-10	.005	0-5 s	Ativa+CR vs Ativa-CR	.156
Ativa+CR vs Sham+CR	.005	0-5 vs 10-15	.001		Ativa+CR vs Sham+CR	.062
Ativa+CR vs Sham-CR	.011	0-5 vs 15-20	.197		Ativa+CR vs Sham-CR	.041
Ativa-CR vs Sham+CR	.066	0-5 vs 20-25	.080		Ativa-CR vs Sham+CR	.190
Ativa-CR vs Sham-CR	.072	0-5 vs 25-30	.122		Ativa-CR vs Sham-CR	.168
Sham+CR vs Sham-CR	.007	5-10 vs 10-15	.006		Sham+CR vs Sham-CR	.017
		5-10 vs 15-20	.214	5-10 s	Ativa+CR vs Ativa-CR	.036
		5-10 vs 20-25	.090		Ativa+CR vs Sham+CR	.224
		5-10 vs 25-30	.123		Ativa+CR vs Sham-CR	.203
		10-15 vs 15-20	.194		Ativa-CR vs Sham+CR	.187
		10-15 vs 20-25	.078		Ativa-CR vs Sham-CR	.167
		10-15 vs 25-30	.122		Sham+CR vs Sham-CR	.013
		15-20 vs 20-25	.136	10-15 s	Ativa+CR vs Ativa-CR	.129
		15-20 vs 25-30	.350		Ativa+CR vs Sham+CR	.017
		20-25 vs 25-30	.226		Ativa+CR vs Sham-CR	.195
					Ativa-CR vs Sham+CR	.125
					Ativa-CR vs Sham-CR	.337
					Sham+CR vs Sham-CR	.235
				15-20 s	Ativa+CR vs Ativa-CR	.000
					Ativa+CR vs Sham+CR	.120
					Ativa+CR vs Sham-CR	.036
					Ativa-CR vs Sham+CR	.114
					Ativa-CR vs Sham-CR	.034
					Sham+CR vs Sham-CR	.159
				20-25 s	Ativa+CR vs Ativa-CR	.171
					Ativa+CR vs Sham+CR	.033
					Ativa+CR vs Sham-CR	.099
					Ativa-CR vs Sham+CR	.150
					Ativa-CR vs Sham-CR	.059
					Sham+CR vs Sham-CR	.074
				25-30 s	Ativa+CR vs Ativa-CR	.122
					Ativa+CR vs Sham+CR	.325
					Ativa+CR vs Sham-CR	.054
					Ativa-CR vs Sham+CR	.218
					Ativa-CR vs Sham-CR	.064
					Sham+CR vs Sham-CR	.267

Note. Ativa+CR = active condition with cognitive reappraisal; Ativa-CR = active condition without cognitive reappraisal; Sham+CR = sham condition with cognitive reappraisal; Sham-CR = sham condition without cognitive reappraisal

Table S12. Respiratory rate - effect size results of three-way mixed-design ANOVA - vmPFC

				Condition x Time		
Condition	Cohen's *d*	Time	Cohen's *d*	Interval	Condition	Cohen's *d*
Ativa+CR vs Ativa-CR		0-5 vs 5-10	.302	0-5 s	Ativa+CR vs Ativa-CR	.019
Ativa+CR vs Sham+CR		0-5 vs 10-15	.260		Ativa+CR vs Sham+CR	.095
Ativa+CR vs Sham-CR		0-5 vs 15-20	.195		Ativa+CR vs Sham-CR	.059
Ativa-CR vs Sham+CR		0-5 vs 20-25	.348		Ativa-CR vs Sham+CR	.115
Ativa-CR vs Sham-CR		0-5 vs 25-30	.202		Ativa-CR vs Sham-CR	.079
Sham+CR vs Sham-CR		5-10 vs 10-15	.061		Sham+CR vs Sham-CR	.035
		5-10 vs 15-20	.109	5-10 s	Ativa+CR vs Ativa-CR	.111
		5-10 vs 20-25	.024		Ativa+CR vs Sham+CR	.168
		5-10 vs 25-30	.111		Ativa+CR vs Sham-CR	.192
		10-15 vs 15-20	.055		Ativa-CR vs Sham+CR	.059
		10-15 vs 20-25	.092		Ativa-CR vs Sham-CR	.062
		10-15 vs 25-30	.055		Sham+CR vs Sham-CR	.006
		15-20 vs 20-25	.141	10-15 s	Ativa+CR vs Ativa-CR	.112
		15-20 vs 25-30	.001		Ativa+CR vs Sham+CR	.165
		20-25 vs 25-30	.145		Ativa+CR vs Sham-CR	.185
					Ativa-CR vs Sham+CR	.019
					Ativa-CR vs Sham-CR	.047
					Sham+CR vs Sham-CR	.040
				15-20 s	Ativa+CR vs Ativa-CR	.086
					Ativa+CR vs Sham+CR	.268
					Ativa+CR vs Sham-CR	.111
					Ativa-CR vs Sham+CR	.180
					Ativa-CR vs Sham-CR	.028
					Sham+CR vs Sham-CR	.144
				20-25 s	Ativa+CR vs Ativa-CR	.226
					Ativa+CR vs Sham+CR	.131
					Ativa+CR vs Sham-CR	.205
					Ativa-CR vs Sham+CR	.080
					Ativa-CR vs Sham-CR	.389
					Sham+CR vs Sham-CR	.299
				25-30 s	Ativa+CR vs Ativa-CR	.096
					Ativa+CR vs Sham+CR	.301
					Ativa+CR vs Sham-CR	.089
					Ativa-CR vs Sham+CR	.264
					Ativa-CR vs Sham-CR	.225
					Sham+CR vs Sham-CR	.457

Note. Ativa+CR = active condition with cognitive reappraisal; Ativa-CR = active condition without cognitive reappraisal; Sham+CR = sham condition with cognitive reappraisal; Sham-CR = sham condition without cognitive reappraisal

Table S13. Positive and Negative Affect (PANAS) before and after the experimental sessions - dlPFC

	**Active dlPFC**						**Sham dlPFC**					
	Pre	Post	Paired *t* test				Pré	Post	Paired *t* test			
	M (*SD*)	*M* (*SD*)	*df*	*t*	*p*	Cohen's *d*	*M* (*SD*)	*M* (*SD*)	*df*	*t*	*p*	Cohen's *d*
Positive affect	32.47 (6.41)	29.16 (7.51)	44	3.97	*p* < .001	.592	33.13 (7.05)	29.07 (7.30)	45	7.06	*p* < .001	1.041
Negative affect	13.33 (3.75)	14.11 (4.58)	44	-1.27	.212	.189	14.96 (5.03)	14.50 (5.47)	45	.69	.507	.099

Table S14. PANAS before and after the experimental sessions - vmPFC

	**Active dlPFC**						**Sham dlPFC**					
	Pre	Post	Paired *t* test				Pre	Post	Paired *t* test			
	M (*SD*)	*M* (*SD*)	*df*	*t*	*p*	Cohen's *d*	*M* (*SD*)	*M* (*SD*)	*df*	*t*	*p*	Cohen's *d*
Positive affect	32.23 (5.86)	27.33 (7.21)	38	5.66	*p* < .001	.905	30.56 (6.66)	27.44 (6.98)	38	4.34	*p* < .001	.696
Negative affect	13.74 (4.70)	13.85 (4.80)	38	-.12	.903	.020	14.28 (3.52)	13.59 (4.84)	38	.84	.407	.134

Table S15. PANAS between active and sham groups - dlPFC and vmPFC

	**dlPFC**						**vmPFC**					
	Active	Sham	*t* test				Active	Sham	*t* test			
	M (*SD*)	*M* (*SD*)	*df*	*t*	*p*	Cohen's *d*	*M* (*SD*)	*M* (*SD*)	*df*	*t*	*p*	Cohen's *d*
Positive affect-Pre session	32.47 (6.41)	33.13 (7.05)	88.54	-.47	.639	.114	32.23 (5.86)	30.56 (6.66)	74.76	1.17	.244	.281
Negative affect-Pre session	13.33 (3.75)	14.96 (5.03)	83.18	-1.75	.084	.300	13.74 (4.70)	14.28 (3.52)	70.46	-.57	.569	.113
Positive affect-Post session	29.30 (7.50)	29.07 (7.30)	89.93	.16	.877	.037	27.33 (7.21)	27.44 (6.98)	75.92	-.06	.949	.021

Table S16. Visual analogue scale for tDCS side effects - dlPFC

	**Active dlPFC**					**Sham dlPFC**				
	Pre	Post	*t* test			Pré	Post	*t* test		
	M (*SD*)	*M* (*SD*)	*df*	*t*	*p*	*M* (*SD*)	*M* (*SD*)	*df*	*t*	*p*
Tiredness	4.42 (2.51)	3.80 (2.93)	44	2.59	.013*	4.22 (2.43)	4.20 (2.66)	45	.07	.944
Anxiety	2.69 (2.47)	2.29 (2.65)	44	1.12	.271	3.17 (2.66)	2.57 (2.67)	45	1.79	.080
Sadness	1.00 (1.41)	1.00 (1.46)	44	.00	1.000	1.46 (2.24)	1.46 (2.18)	45	.00	1.00
Agitation	1.89 (2.10)	2.07 (2.30)	44	-.45	.658	2.13 (2.39)	2.30 (2.67)	45	-.50	.621
Sleepiness	4.04 (2.90)	3.58 (3.10)	44	1.43	.160	3.96 (2.70)	3.80 (2.73)	45	.55	.587
Itch	.22 (.60)	.96 (2.28)	44	-2.10	.041*	.22 (.55)	.63 (1.44)	45	-1.97	.055
Headache	.76 (1.68)	.73 (1.42)	44	.12	.908	.72 (1.36)	1.02 (1.69)	45	-1.27	.212
Another pain	.47 (1.58)	.36 (1.23)	44	1.04	.302	.46 (1.13)	.20 (.62)	45	2.48	.017*
Anthill	.27 (1.51)	.33 (1.52)	44	-1.14	.261	.26 (1.08)	.50 (1.36)	45	-.97	.338
Metallic taste	.07 (.33)	.13 (.66)	44	-.72	.473	.13 (.62)	.15 (.89)	45	-.13	.894

Note. *$p < .05$

Negative affect-Post session	14.20 (4.57)	14.50 (5.47)	87.24	-.29	.773	.059	13.85 (4.80)	13.59 (4.84)	75.99	.24	.815	.064

Table S17. Visual analogue scale for tDCS side effects - vmPFC

	**Active vmPFC**					**Sham vmPFC**				
	Pre	Post	*t* test			Pre	Post	*t* test		
	M (*SD*)	*M* (*SD*)	*df*	*t*	*p*	*M* (*SD*)	*M* (*SD*)	*df*	*t*	*p*
Tiredness	3.74 (2.54)	3.92 (2.60)	38	-.49	.628	4.15 (2.44)	4.03 (2.88)	38	.47	.638
Anxiety	2.92 (3.08)	2.41 (2.75)	38	1.42	.164	3.10 (2.47)	2.10 (2.41)	38	3.04	.004**
Sadness	1.00 (1.49)	.95 (1.69)	38	.18	.857	1.26 (1.89)	1.13 (1.98)	38	.67	.507
Agitation	1.95 (2.29)	1.87 (2.38)	38	.21	.831	2.31 (2.23)	2.26 (2.65)	38	.15	.883
Sleepiness	3.10 (2.72)	3.31 (2.94)	38	-.63	.532	3.87 (3.05)	3.49 (3.96)	38	1.39	.172
Itch	.62 (1.37)	1.18 (1.90)	38	-1.92	.062	.15 (.81)	.49 (1.54)	38	-1.22	.230
Headache	.59 (1.37)	.49 (1.45)	38	.36	.722	.74 (1.58)	.90 (1.85)	38	-.55	.587
Another pain	1.05 (2.46)	.62 (1.63)	38	1.32	.195	.33 (1.34)	.21 (1.13)	38	1.96	.058
Anthill	.03 (.16)	.18 (.60)	38	-1.53	.136	.03 (.16)	.08 (.35)	38	-.81	.421
Metallic taste	.05 (.22)	.00 (.00)	38	1.43	.160	.05 (.32)	.15 (.67)	38	-1.28	.210

Note. ** $p < .01$
